# Raman Microspectroscopy for Structural Indication in Ultrafast Laser Writing

**DOI:** 10.1002/smtd.202502413

**Published:** 2026-03-15

**Authors:** Xingrui Cheng, Eugenio Picheo, Zhixin Chen, Martin J. Booth, Patrick S. Salter, Álvaro Fernández‐Galiana

**Affiliations:** ^1^ Department of Engineering Science University of Oxford Oxford UK; ^2^ Department of Materials University of Oxford Oxford UK

**Keywords:** diamond electrodes, diamond graphitization, femtosecond laser writing, in‐situ metrology, Raman microspectroscopy

## Abstract

Femtosecond laser fabrication enables the creation of a wide range of devices, but its scalability and yield can be limited by the lack of real‐time, in situ monitoring tools. In particular, there is a strong need for metrics that directly correlate with device performance. Raman microspectroscopy provides a non‐destructive route for in situ characterization. Here, we demonstrate its potential to assess the electrical performance of laser‐written graphitic electrodes in diamond. By combining hyperspectral mapping with electrical testing, we show that depletion of the 1332 ${\mathrm{cm}}^{-1}$ sp3 Raman line serves as a monotonic and robust predictor of resistance, offering clear advantages over commonly used spectral features. We further introduce hyperspectral unmixing as a label‐free approach to identify relevant spectral signatures in fabrication processes where Raman markers are less defined. Importantly, the methodology we present is not restricted to diamond but can be adapted to other host materials and functionalities, offering a practical path toward specification‐driven fs‐laser microfabrication.

## Graphical abstract

Raman microspectroscopy is demonstrated as an in situ, phase‐specific probe for femtosecond laser fabrication in diamond. Multiple spectral indicators are systematically evaluated and correlated with electrical performance, establishing a robust methodology for process monitoring. This framework lays the groundwork for future feedback‐integrated fabrication strategies and is readily transferable to other materials and laser‐written device platforms.

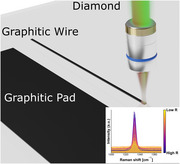

## 1 Introduction

Femtosecond laser writing enables direct, 3D patterning of embedded features in transparent hosts via nonlinear absorption–multi‐phonon ionization and Zener breakdown–that initiates localized structural transformation [1, 2]. The technique has found particular application in processing of diamond [3, 4, 5, 6, 7], permitting fabrication of buried and surface carbon wires and electrode architectures with arbitrary geometry for electrically conductive devices [8], such as radiation detectors [9, 10].

Yet, one of the main current limitations for scaling manufacturing of these laser‐written devices is the lack of feedback mechanisms that can be integrated for real‐time, in‐situ process monitoring and control. Indeed, most current structural analyses remain destructive or ex situ (e.g., TEM [11, 12], SEM [5], and X‐ray diffraction microscopy [13]).

Raman microspectroscopy, based on inelastic light scattering, has been extensively used for non‐destructive identification, structural characterization, and monitoring of chemical and physical properties across materials science, chemistry, and biology [14].

In recent years, significant efforts have also focused on developing advanced computational approaches for spectral analysis, further enhancing and broadening its range of applications.

Given its ability to differentiate the spectral signatures of sp3 versus sp2 bonding, crystallinity, and local strain environment, Raman spectroscopy is ideal for non‐invasive, in situ, spatially resolved characterization of carbon systems [15, 16]. In carbon systems, Raman signatures provide direct markers of bonding and structural order: graphitic sp2 domains give rise to the G band near $∼\;1580\;{\mathrm{cm}}^{-1}$, while disorder or finite crystallite size activates the D band at $∼\;1350-1360\;{\mathrm{cm}}^{-1}$ [17, 18]. In contrast, single‐crystal diamond exhibits a sharp first‐order mode at $1332\;{\mathrm{cm}}^{-1}$ [19, 20]. Throughout this work, we denote these features as the sp3 line ($1332\;{\mathrm{cm}}^{-1}$), sp2 band ($∼\;1580\;{\mathrm{cm}}^{-1}$), and D band ($∼\;1350-1360\;{\mathrm{cm}}^{-1}$), providing a consistent basis for assessing carbon phase composition, crystallinity, and defect populations in laser‐written structures.

Here, we use Raman microspectroscopy to systematically assess fs‐laser‐written graphitic electrodes in diamond, and compare its performance with brightfield and photoluminescence imaging. Leveraging phase‐specific vibrational modes of carbon, we perform direct mapping of surface graphitization through explicit band assignment and label‐free spectral deconvolution, and correlate the spectral signatures to the device performance (i.e., electrical conductivity). Beyond offering detailed insights into graphitization monitoring in diamond, the methodology outlined here is broadly transferable: it can be applied to evaluate Raman spectroscopy as a performance probe in diverse device platforms, since none of its principles are inherently diamond‐specific.

## 2 Results and Discussion

**FIGURE 1:**
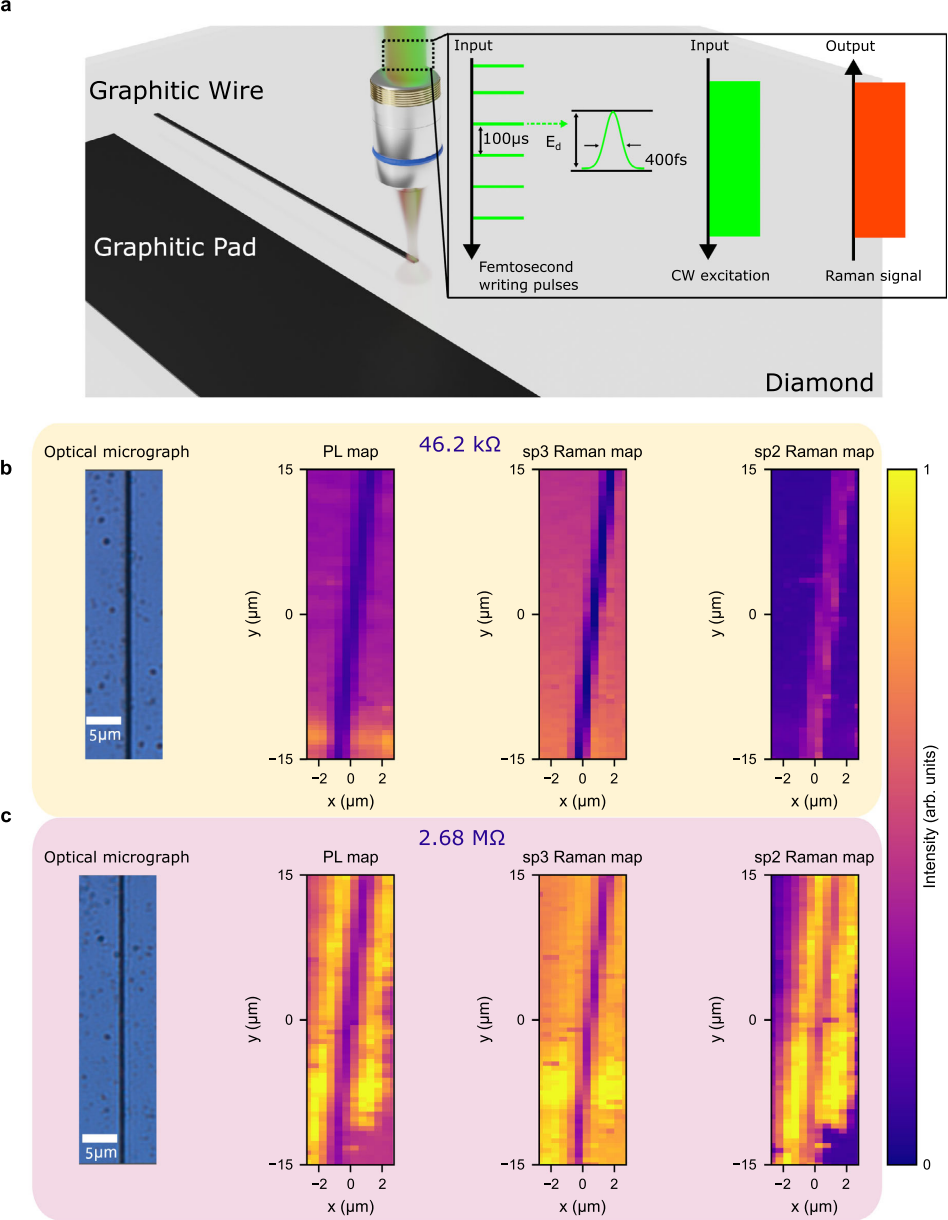
Laser‐written graphitic structures with optical/PL/Raman characterization. a) Schematic of fs‐laser fabrication of graphitic pads and wires. The fabrication beam shares a common optical path with the 532 nm Raman excitation, and the back‐scattered Raman signal is collected to enable in situ monitoring and post‐fabrication mapping. b–c) Optical micrographs, PL maps, and Raman intensity maps (sp3 and sp2 windows) for wires written at scan speeds of 20 and 200 μm $s^{-1}$, respectively, collected after fabrication. For direct comparison, PL and Raman maps are unprocessed and share identical count limits. The 20 μm $s^{-1}$ wire exhibits a resistance of 46.2kΩ, whereas the 200 μm $s^{-1}$ wire exhibits 2.68MΩ. The stronger optical darkening at the lower scan speed provides a first qualitative indication of increased graphitization. However, the contrast in optical micrographs and PL maps alone is difficult to resolve quantitatively. By contrast, Raman mapping of the sp3 window provides a direct and more reliable measure of the degree of graphitization, offering phase‐specific insight that complements the broadband PL response and sp2 contrast.

To model the principal conductive elements in diamond devices, we use a femtosecond laser to fabricate graphitic pads and wires on the surface of a CVD diamond sample, as shown in Figure 1a. Pads wider than 25 and 200 μm long reproduce the large‐area contact zones required for wire bonding or subsequent metallization, where sheet resistance and adhesion set the performance limit. By contrast, 1 μm × 200 μm wires replicate the sub‐micron buried interconnects already deployed in high‐density pixel detectors, biosensing micro‐electrodes, and microwave striplines for NV‐center control [12, 21, 22]. Studying both geometries spans the full electrical landscape of laser‐modified diamond.

Moreover, pads and wires of equal length were fabricated at varying laser scanning speeds to produce different degrees of graphitization, and thus a controlled range of electrical conductivities.

For each of these structures, brightfield transmission microscopy images were captured, along with photoluminescence (PL) and backscattered Raman, collected using our integrated confocal microscope (see Materials and Methods). In all cases, PL and Raman measurements were performed using excitation powers verified to be below the threshold for laser‐induced modification, ensuring that the imaging process did not introduce additional damage or alter the local degree of graphitization.

Figure 1b,c show these optical micrographs along with PL and Raman maps (sp3 and sp2) for two laser‐written graphitic wires fabricated at scan speeds of 20 and 200 μm $s^{-1}$. The wire written at 20 μm $s^{-1}$ exhibits a resistance of 46.2kΩ, whereas the 200 μm $s^{-1}$ wire shows 2.68MΩ, corresponding to an ∼58× difference (see Figure 4b; Note S7 for complete set of conductivity measurements). Details of the graphitic pads are provided in Note S1. Under optical illumination, the laser‐written track appears dark relative to the surrounding single‐crystal diamond. This contrast arises because conversion from transparent, wide‐band‐gap sp3 diamond to disordered sp2 carbon markedly increases visible‐wavelength absorption and slightly raises the refractive index, thereby reducing transmittance through the modified region [23, 24]. Therefore, the degree of darkening can be used as a first qualitative indication of graphitization. However, it conflates absorption, scattering, debris, and illumination artifacts, and it is not phase‐specific. For this reason, PL and hyperspectral Raman mapping are regarded as more reliable approaches, since they provide phase‐specific, quantitative, and spatially resolved indicators of graphitization.

**FIGURE 2:**
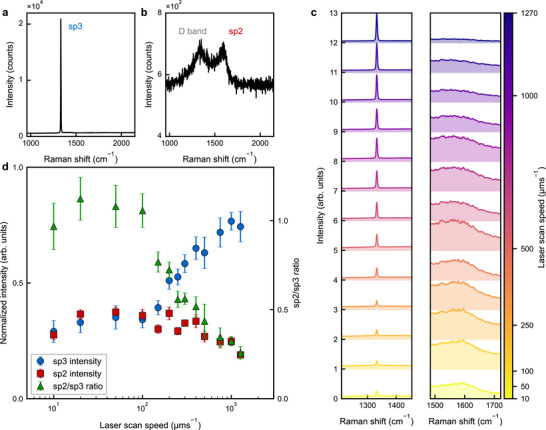
Spectroscopic analysis of laser‐written graphitic structures at different scan speeds. a,b) Representative normalized Raman spectra (532 nm excitation) from pristine diamond and a fully graphitized region (see Materials and Methods and Note S2 for normalization and pixel selection). The pristine diamond spectrum is dominated by the sp3 line at 1332 ${\mathrm{cm}}^{-1}$. In the graphitized region the sp3 signal is strongly suppressed, while a broad disorder‐activated D band appears near 1350 ${\mathrm{cm}}^{-1}$ together with the sp2 G peak centered around 1580 ${\mathrm{cm}}^{-1}$, indicative of local graphitization. c) Scan‐speed‐dependent mean Raman spectra restricted to the sp3 (diamond) and sp2 (graphitic) windows, shown for wires written at 10–1270 μm $s^{-1}$ (top to bottom). For each speed, the mean is computed from 60 spectra per wire extracted from the hyperspectral maps, revealing progressive depletion of sp3 features and growth of the sp2 response with decreasing laser scan speed. d) Speed‐dependent quantitative summary: integrated intensities (from 60 spectra per wire extracted from the hyperspectral maps) of the sp3 and sp2 windows, together with the sp2/sp3 ratio, plotted against scan speed, error bar indicating one sigma error.

**FIGURE 3:**
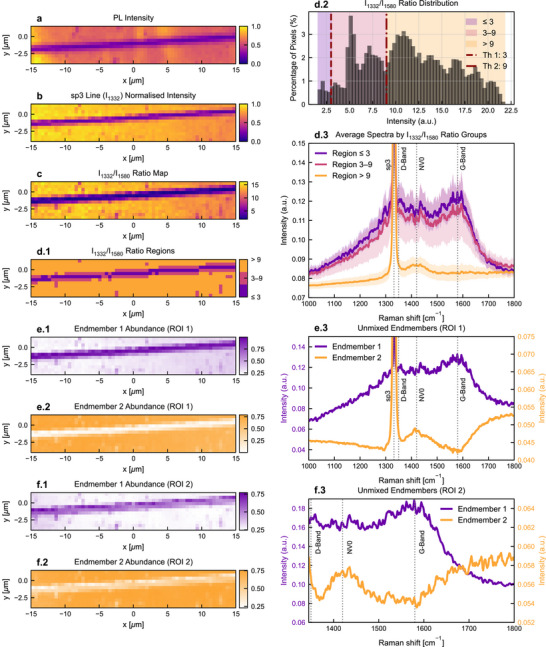
Evaluation of spectral unmixing for electrode surface graphitization. a,b) PL intensity map and normalized first‐order Raman intensity (sp3 line at 1332 ${\mathrm{cm}}^{-1}$) for a graphitic wire written at 50 μm $s^{-1}$ (74.8 kΩ). c) Map of sp3‐to‐sp2 ratio. d) Threshold‐based segmentation by the sp3‐to‐sp2 ratio $\rho =I_{1332}/I_{1580}$. (d.1) Segmentation map using experimentally determined thresholds for non‐graphitized (ρ≤3), partially graphitized (3<ρ<9), and fully‐graphitized (ρ≥9). (d.2) Pixel distribution of $\rho_{sp3}$ over the complete set of graphitic wires spectral data (i.e., including all different writing speeds), depicting selected thresholds. (d.3) Class‐averaged spectra (mean ± 1 s.d.) for the 74.8 kΩ electrode, showing the sp3 line (1332 ${\mathrm{cm}}^{-1}$), D band (∼1350 ${\mathrm{cm}}^{-1}$), ${\mathrm{NV}}^{0}$ fluorescence (∼1420 ${\mathrm{cm}}^{-1}$), and G band (∼1580 ${\mathrm{cm}}^{-1}$). e) Spectral unmixing by Vector Component Analysis (VCA) with Fully Constrained Least Squares (FCLS) over spectral region of interest (ROI) 1 (1000–1800cm^−1^): FCLS abundance maps for endmember 1 (e.1) and endmember 2 (e.2), and corresponding endmember spectra with band markers (e.3). f) Same unmixing workflow over ROI 2 (1345–1800 ${\mathrm{cm}}^{-1}$, excluding the 1332 ${\mathrm{cm}}^{-1}$ line): abundance maps for endmember 1 (f.1) and endmember 2 (f.2), and endmember spectra (f.3).

**FIGURE 4:**
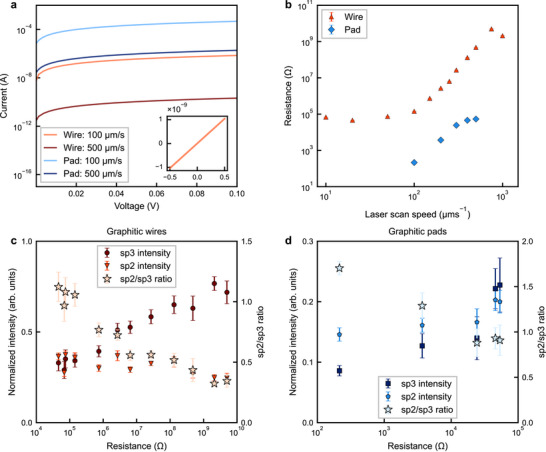
Direct Raman‐electrical correlation of fs‐laser‐induced graphitization. a) Semi‐log I–V curves from 0 to 100 mV for pads and wires written at scan speeds of 100 and 500 μm $s^{-1}$ (100 μm $s^{-1}$: light red = wire, light blue = pad; 500 μm $s^{-1}$: dark red = wire, dark blue = pad). Inset: linear‐scale I−‐V for a representative wire over −500 to +500 mV, showing symmetric ohmic behavior. Pad electrodes generally exhibit lower resistance than wires, attributable to larger cross‐sections and a higher effective degree of graphitization. b) Resistance of laser‐written pads and wires versus laser scan speed. Slower scan speeds yield lower resistance. Values are obtained from linear least‐squares fits to *I*‐*‐V* data acquired within ±500 mV in a two‐probe configuration (see Electrical conductivity measurement), error bar indicates one sigma error. Of note, at the lowest scanning speeds, wire resistance is comparable to that of the contact electrodes, reducing measurement accuracy (see 4). Also, the wire written at 750μms 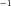 exhibits non‐ohmic I–V behavior (see Note S10), giving a higher apparent resistance than the wire written at 1000μms 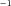. c,d) Resistance dependence of the mean normalized Raman intensities in the sp3 (diamond) and sp2 (graphitic) windows, as well as the sp2 to sp3 ratio, is shown.

In the PL maps (Figure 1b,c), the laser‐written graphitized regions appear dark because the processing introduces non‐radiative recombination pathways that quench the broadband fluorescence. Yet, it is not phase‐specific and can be easily affected by broadband emission, as shown in Figure 1c and detailed in Note S2. On the other hand, Raman mapping provides phase‐specific contrast.

Local graphitization converts sp3 diamond to sp2 carbon, depleting the sp3 line and enhancing the G band; accordingly, we construct sp3 maps by integrating 1325–1340 ${\mathrm{cm}}^{-1}$ and sp2 maps by integrating 1575–1610 ${\mathrm{cm}}^{-1}$ [23, 25]. These phase‐specific features (sp3 line, sp2 band, D band) enable quantitative, cross‐structure comparison of phase content, crystallinity, and defect populations in laser‐written carbon [11, 16, 25]. Note that, as shown in (Figure 1c), Raman signal can also be contaminated by non‐specific fluorescent background, an effect that can oftentimes be minimized with baseline correction and other techniques [26].

### 2.1 Spectral Characterization of Laser‐Written Electrodes

As shown in Figure 1, one of the limitations of both brightfield microscopy and broadband PL is that despite being good techniques for qualitative assessment of the fabrication they present limitations when used to provide phase‐specific quantitative information. In the case of PL, it can in principle be used for quantitative sp3‐to‐sp2 conversion evaluation, but its sensitivity to broadband emission not stemming from the process of interest reduces its fidelity. This is further discussed in Notes S3 and S4. Therefore, we assessed the suitability of Raman spectroscopy for this task.

In graphitic carbons, the in‐plane first‐order $E_{2g}$ phonon (“G band”) appears near $∼\;1580\;{\mathrm{cm}}^{-1}$, while disorder or finite crystallite size activates the defect‐induced “D band” around $∼\;1350-1360\;{\mathrm{cm}}^{-1}[$ 17, 18]. In single‐crystal diamond, the zone‐center optical phonon of $F_{2g}$ symmetry gives a sharp first‐order line at $1332{\mathrm{cm}}^{-1}[$ 19, 20].

These emphasize the phase origin of each peak and facilitate quantitative comparison across laser‐written structures, enabling identification and quantification of carbon phases, crystallinity, and defect populations [11, 16, 25].

#### 2.1.1 Single‐Band Characterization

Using the integrated confocal microscope, we collected hyperspectral Raman maps on every graphitic wire and pad fabricated at distinct scan speeds (see Materials and Methods). Figure 2a,b display representative spectra for (a) the unmodified single‐crystal diamond and (b) a fully graphitized pad fabricated at a laser scan speed of 100 μm $s^{-1}$. The pristine crystal is dominated by the sp3 line at 1332 ${\mathrm{cm}}^{-1}$. In contrast, the graphitic structure written with low laser scan speed exhibits depleted sp3 intensity and is governed by the disorder‐induced D band (1350 ${\mathrm{cm}}^{-1}$) together with the sp2 band (graphitic G band (1580 ${\mathrm{cm}}^{-1}$)), evidencing almost complete conversion to sp2 carbon.

Figure 2c compiles the speed‐dependent averaged spectra of the laser‐written graphitic wires. The spectral data normalized across all the samples to the sp3 peak (left panel) and the sp2 band (right panel), allowing the two regimes to be compared on equal footing across writing speeds. At the highest velocity tested (500 μm $s^{-1}$) the spectrum is sp3‐dominated and the D and G bands are barely discernible, implying that only a small fraction of lattice sites have been converted to sp2. Progressively reducing the speed, and thereby increasing the deposited energy density per unit length, reverses this balance: the D and sp2 bands intensify while the sp3 line diminishes, signaling enhanced graphitization. The evolution is summarized quantitatively in Figure 2d, which plots the normalized integrated intensities $I_{\mathrm{sp}3}$ and $I_{\mathrm{sp}2}$ as well as their ratio $I_{\mathrm{sp}2}/I_{\mathrm{sp}3}$ versus laser scan speed. $I_{\mathrm{sp}3}$ depletes with decreasing speed, whereas $I_{\mathrm{sp}2}$ increases, except for a modest downturn below 50 μm $s^{-1}$, where excessive dose does not yield higher sp2 band intensity.

#### 2.1.2 Multi‐Band Characterization

Laser‐written electrodes in diamond contain both unconverted sp3‐bonded lattice and newly formed sp2‐bonded carbon. Within a confocal voxel, these phases typically coexist as sub‐micron domains, so a single Raman spectrum is an average over a heterogeneous local volume.

In the previous section, we showed that the degree of graphitization can be inferred from the intensity of certain bands (e.g., sp3 line). However, relying on a single peak can be fragile in practice because the apparent intensity can be influenced by various factors such as laser power fluctuations, defocusing, local topography and debris, baseline fluorescence, or strain‐induced shifts/broadening.

To mitigate these effects, ratio metrics that combine several informative bands are usually preferred over single‐peak readouts. In our case the most natural choice is to use the sp2/sp3 ratio, containing the information carried by the G/D complex and the 1332 ${\mathrm{cm}}^{-1}$ diamond line. Figure 3 illustrates this for a representative wire written at 50 μm $s^{-1}$.

#### 2.1.3 Hyperspectral Unmixing

While ratio maps remove much of the acquisition variability, they still require manual selection of specific bands. Therefore, we also introduce the use of hyperspectral unmixing as a label‐free alternative for fabrication processes where the bands of each phase might not be known.

As described in Materials and Methods, we model each spectrum as a linear mixture of n endmembers and use well‐established linear unmixing methods, such as vector component analysis (VCA) [27] to estimate these endmembers directly from the data. Then, abundances are estimated per pixel solving the linear inverse problem. In particular, for the results shown in Figure 3, we impose n=2 endmembers and enforce both non‐negativity, and the sum‐to‐one of the abundances at every pixel using fully constrained least squares (FCLS) [28], which enables a direct fractional interpretation without the need for external references.

As shown in Figure 3e, when applied over a broad region of interest (1000–1800${\mathrm{cm}}^{-1}$) unmixing yields endmembers that, while not identical to the actual reference spectra presented in Figure 3), preserve the expected relevant features: the diamond‐associated endmember is characterized by a sharp 1332 ${\mathrm{cm}}^{-1}$ line, whereas the graphite‐associated endmember exhibits a broad D/G complex with a suppressed diamond line. Additionally, the diamond‐associated endmember also features a broader band centered around ∼1430 ${\mathrm{cm}}^{-1}$, which is consistent with the zero‐phonon line of neutral single nitrogen‐vacancy defects (${\mathrm{NV}}^{0}$) in diamond [29]. The resulting abundance maps also closely track the simpler sp3‐window intensity maps (Figure 3b).

To test sensitivity to the dominant diamond line, we repeated the analysis after excluding 1332 ${\mathrm{cm}}^{-1}$ (spectral ROI = 1345–1800 ${\mathrm{cm}}^{-1}$). In this case, the diamond‐associated endmember is defined primarily by the ${\mathrm{NV}}^{0}$ contribution within the spectral ROI, while the complementary endmember remains the broad G/D complex. The corresponding abundance maps retain the expected spatial structure but exhibit lower signal‐to‐noise (SNR), as anticipated when the most intense, phase‐specific line is removed.

Overall, the unmixing results reinforce the interpretation from ratio maps while removing the need to hand‐pick bands: knowing only that two materials are present, the algorithm discovers representative endmembers and delivers per‐pixel abundance maps that agree with the PL/Raman contrasts.

### 2.2 Raman Monitoring of Electrical Performance

Ultimately, the optimal feedback signal for in situ monitoring of fabrication should be directly tied to the functional performance of the resulting devices. For graphite electrodes on diamond, this performance is best represented by the conductivity of the laser‐written features. To this end, the electrical conductivity of the pads and wires was measured using a custom setup (see Materials and Methods).

Figure 4 presents our findings and consolidates the link between laser scan speed, local bonding state, and device‐level conductivity of the laser‐written features. Figure 4a depicts the current–voltage traces for all wires and pads, which are linear over ±500 mV, indicating ohmic behavior and no measurable Schottky barriers; conduction is therefore governed by the volume fraction and connectivity of the laser‐generated graphitic phase. For the 200 μm‐wide graphitic structure, written at 100μms 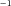, the pad resistance is 332Ω, versus $1.46\times 10^{5}\;\Omega$ for the wire at the same speed. The lower pad resistance reflects its larger cross‐section and additional over‐writing during fabrication, which further graphitizes the diamond and increases the graphite fraction. All resistances are extracted from linear fits to quasi‐static I–V sweeps within ±500 mV (details of the IV curves are shown in Notes S10 and S11).

In Figure 4c,d we plot, separately for wires and pads, the mean normalized integrated sp3 and sp2 intensities as well as their ratio versus laser scan speed. As the scan speed increases, the resistance rises concurrently with the normalized sp3 intensity, indicating reduced graphitization and weakened conductive pathways. For wires (Figure 4c), decreasing the scan speed from high to intermediate increases the normalized sp2 intensity and lowers the resistance. However, once the sp3 contribution is strongly diminished (deeply graphitized regime), sp2 intensity alone becomes a less reliable measure of further conductivity enhancement. Since the sp2 intensity rises as the sp3 intensity decreases with scan speed, their ratio mirrors these trends. Pads show the same qualitative behavior (Figure 4d) but remain systematically more conductive than wires at identical scan speeds.

### 2.3 Discussion

Figure 2 summarizes how the Raman features evolve with writing dose, controlled by scan speed. Reducing the speed, which increases the energy deposited per unit length, produces a monotonic depletion of the 1332 ${\mathrm{cm}}^{-1}$ sp3 line and a general rise of the ∼1580 ${\mathrm{cm}}^{-1}$ sp2 band, consistent with progressive diamond‐to‐graphite conversion. At the slowest speeds, however, the sp2 intensity reaches a maximum and then declines despite continued sp3 depletion, a behavior also reported in other studies on diamond graphitization [30, 31].

This counter‐intuitive reduction in sp2 intensity can be interpreted within the “amorphization trajectory” framework of Ferrari et al. [18, 20], wherein progressive disorder drives a transition from nanocrystalline graphite to amorphous sp2‐rich carbon and, ultimately, to highly disordered or tetrahedral amorphous forms. In this regime, shrinking, and fragmenting sp2 clusters suppress well‐defined Raman modes, so the apparent sp2 signal diminishes even as the actual sp2 bonding fraction remains high. At intermediate doses, ordered sp2 clusters grow, strengthening the D and G bands. At extreme doses, however, high defect densities and reduced cluster sizes suppress well‐defined vibrational modes, broadening and merging the D and G peaks into a low‐contrast background. The resulting spectral degradation reduces the apparent sp2 Raman intensity, even when the actual sp2 bonding fraction remains high. Therefore, under such high‐dose conditions, sp3 depletion provides a more reliable metric for tracking graphitization progression, as it decreases monotonically with increasing laser dose, is insensitive to spectral contrast loss, and, thanks to its higher SNR, is less sensitive to non‐specific broadband background [24, 30].

Pad writing shows stronger sp3 depletion than single‐pass wires, which we attribute to stress‐assisted graphitization during overwriting. Lattice mismatch generates tensile stress in the transformed layer and in‐plane compressive stress in adjacent diamond [24]. Compression lowers the graphitization barrier, so overlapping passes enhance conversion. The observed wavenumber shift of the diamond line is consistent with established stress coefficients [32, 33, 34] (see Note S6) and explains the lower resistance measured for pads at identical scan speeds.

In Figure 3, we introduce the use of more advanced Raman‐based structure‐characterization metrics. Particularly we first present multi‐band metrics, mainly sp3‐to‐sp2 ratio. Using this ratio has the advantage of being agnostic to some of the effects that can generate non‐structure‐specific intensity fluctuations in the sp2 and sp3 peaks, such as excitation power. Moreover, we also introduce hyperspectral unmixing as a label‐free complementary spectral analysis (Figure 3). A linear mixing model with non‐negativity and sum‐to‐one constraints recovers two data‐driven endmembers that map onto diamond‐like and graphite‐like responses, producing phase‐fraction maps that agree with band‐based indicators while leveraging the full spectrum. The approach is advantageous as it does not rely on pre‐established bands of interest and is also robust to power drift and can potentially help reduce the effect of baseline fluorescence, though the linearity assumption presents some limitations (see Materials and Methods). Yet, in the case of monitoring graphitization in diamond, where the bands of interest are well‐known, hyperspectral unmixing does not provide a significant advantage.

Finally, in Figure 4 we show how Raman spectroscopy can be used to evaluate the performance of graphite electrodes.

Across devices, depletion of the 1332 ${\mathrm{cm}}^{-1}$ sp3 line correlates with resistance more robustly than absolute sp2 intensity. This is likely due to a combination of the sensitivity of sp2 to disorder in high‐dose conditions and the effect of the broadband background from nongraphitic damage, which is more severe in sp2 in the low‐dose regime due to the difference in SNR with the sp3 line (Figure 1; Note S2).

As shown in Figure 4c,d, the sp2‐to‐sp3 ratio is a useful indicator of graphitization because relative normalization reduces sensitivity to power fluctuations. Yet, under high‐dose irradiation, sp3 exhibits less variance than sp2‐to‐sp3 ratio, likely due to the effect of extreme disorder on the sp2 band [24, 30].

## 3 Conclusion and Outlook

Raman microspectroscopy provides a phase‐specific, quantitative readout that links to ultrafast laser writing conditions and device performance in diamond. Across surface pads and wire electrodes, depletion of the 1332 ${\mathrm{cm}}^{-1}$ sp3 line predicts conductivity more reliably than absolute sp2 and PL intensities. Some of these deficiencies could be solved via spectral pre‐processing, which has the drawback of adding complexity, time, and additional sources of error [35].

Overall, the presented post‐fabrication results indicate that a self‐referenced sp3 metric, benchmarked to the local pristine background, is a robust in situ indicator, while the sp2/sp3 ratio remains useful at moderate doses since it is less sensitive to changes in experimental conditions.

The strong correlation demonstrated in our in situ, post‐fabrication characterization between Raman‐derived metrics and electrical conductivity provides a clear pathway toward real‐time feedback architectures.

In an integrated configuration, the Raman excitation could be spatially offset from the fabrication beam and operated with sub‐second integration times, enabling process monitoring without interrupting fabrication.

Additionally, as shown in Note S2, the excitation beam would contribute to removing surface debris.

As part of this work, linear hyperspectral unmixing has been introduced as a full‐spectrum, label‐free alternative for post‐fabrication mapping and indicator validation. In the case of diamond surface graphitization, unmixing performs analogously with band‐based metrics, demonstrating its potential for samples with less defined structure‐specific bands.

The methodology presented in this work is not specific to diamond structure indicators and could therefore be adapted to other hosts and functionalities (e.g., fabrication in glasses and wide‐bandgap crystals [36, 37, 38], laser‐induced graphene on polymers [39], and embedded heaters or electrodes in SiC, sapphire, silica, and ceramics [40, 41, 42]), providing a practical route toward specification‐driven fs‐laser microfabrication. The suitability of the fabricated structures for device implementation should ultimately be evaluated against application‐specific performance criteria and may require complementary characterization beyond the techniques employed in this study.

## 4 Materials and Methods

### 4.1 Electrode Fabrication

A single‐crystal, optical‐grade ⟨100⟩ diamond grown by chemical‐vapor deposition (Element Six; nominal N concentration <1 ppm) served as the substrate for this study. Surface graphitic pads and wires were inscribed with a fs laser operating at 520 nm ($\tau_{p}=400$ fs, $f_{\text{rep}}=10$ kHz,) and a pulse energy of 30 nJ, using various laser scan speed. Wave‐front aberrations were corrected by a liquid‐crystal spatial light modulator, and the beam was focused through an objective lens with numerical aperture NA=0.50 (ZEISS Plan‐Neofluar, 0.5NA, 20x). Wire electrodes were written in a single pass under continuous stage translation (no overwriting). Successive pulses partially overlapped to form a continuous track; at fixed $f_{\text{rep}}$ the pulse‐to‐pulse separation was set by the scan speed. Pad electrodes were produced by stacking parallel wires with a constant 0.5 μm pitch, maintained for all scan speeds.

### 4.2 Electrical Conductivity Measurement

The resistance of laser‐written pads and wires was determined from two‐probe current–voltage (*I*–*V*) measurements using a low‐noise transimpedance amplifier (DLPCA‐200, FEMTO) together with a real‐time voltage source and data‐acquisition controller (ADwin‐Gold II, Jäger). Electrical contact was made with two micromanipulated Pt probes. For pad devices, the probes contacted opposite ends of each laser‐written pad. For wire devices, both termini were first connected to laser‐written square pads (50 μm × 50 μm, written at 100 μm $s^{-1}$), and the probes landed on these pads to standardize the contact area. Prior to electrical measurements, the sample surface was gently wiped using lens tissue wetted with isopropyl alcohol to remove debris generated during laser‐induced graphitization. The ADwin generated quasi‐static voltage sweeps within the ohmic window (typically ±500 mV) and synchronously digitized the DLPCA‐200 output.

### 4.3 Brightfield Imaging

Optical micrographs were captured with an integrated transmission optical microscope. A red‐LED array situated under the sample serves as illumination source and the images are captured on a CCD camera (EC650, Prosilica Inc.).

### 4.4 Confocal Imaging

Our fabrication setup had an integrated custom confocal microscope that could be simultaneously utilized for photoluminescence and Raman spectral collection. Using a 532 nm continuous‐wave excitation laser (Cobolt Samba 150mW) and a fast steering mirror (FSM‐300, Newport) relayed via a 4f into the back aperture of the system's objective, 2D photoluminescence and Raman maps could be collected. Notably, in this study, the 2D maps were collected post‐fabrication utilizing a higher numerical aperture objective (Olympus PlanApo 0.95NA, 60x).

Both photoluminescence and Raman spectra were collected simultaneously employing 30 mW of laser excitation. All hyperspectral maps in this work were acquired after fabrication, serving to validate indicators intended for real‐time implementation. The samples were scanned with a 500nm pixel‐to‐pixel lateral displacement and a 1 s exposure time. The collected signal was split 99:1 between the Raman and the photoluminescence collection paths. For the latter, signal was collected via a single photon avalanche detector (SPCM‐AQRH‐14‐FC, Excelitas Inc.). Given that the collection path of our confocal setup features a 550 nm long‐pass dichroic, and that the SPAD quantum efficiency drops significantly after 850 nm, the PL collection window can be considered 550–850 nm. For the Raman collection path, scattered light was dispersed by a 1200 lines ${\mathrm{mm}}^{-1}$ blazed spectrograph (SpectraPro HRS‐1200) and detected on a deep‐cooled CCD camera (PIXIS 100, Princeton Instruments).

### 4.5 Raman Spectra Preprocessing

The hyperspectral Raman data were loaded as a 3D array I(x,y,λ). To enable cross‐dataset comparison (accounting for sample tilt, focus drift, and excitation‐power fluctuations), we pre‐normalized each map using pristine diamond regions identified from the sp3 window. Specifically, we integrated I(x,y,λ) over [1325,1340] ${\mathrm{cm}}^{-1}$ to obtain $I_{\mathrm{int}}\left(i,j\right)$, ranked all pixels, and defined the $N_{\mathrm{bright}}$ brightest pixels (wire electrodes: $N_{\mathrm{bright}}=200$; pad electrodes: $N_{\mathrm{bright}}=1000$) as the pristine reference. The normalization factor $F_{\mathrm{norm}}$ was the mean of these $I_{\mathrm{int}}$ values, and the hyperspectral cube was normalized as $I_{\mathrm{norm}}\left(i,j,\lambda \right)=I\left(i,j,\lambda \right)/F_{\mathrm{norm}}$, establishing $\langle I^{\mathrm{norm}}\mathrm{sp}3\rangle \mathrm{pristine}=1$.

From $I_{\mathrm{norm}}$, we computed integrated intensities for sp3 (diamond, [1325,1340] ${\mathrm{cm}}^{-1}$) and sp2 (graphitic, [1575,1610] ${\mathrm{cm}}^{-1}$) at each pixel and identified $N_{\mathrm{dark}}$ darkest sp3 pixels (60 for wire and 1500 for pad) as modified regions. For these pixels, we report the spatial coordinates, the normalized integrals $I^{\mathrm{norm}}\mathrm{sp}3$ and $I^{\mathrm{norm}}\mathrm{sp}2$, their averages, and the ratio $R=I^{\mathrm{norm}}\mathrm{sp}2/I^{\mathrm{norm}}\mathrm{sp}3$ as a quantitative metric of graphitization. Fixed integration windows were used throughout (sp3 width 15 ${\mathrm{cm}}^{-1}$; sp2 width 35 ${\mathrm{cm}}^{-1}$); thus, the sp2/sp3 ratio was the ratio of integrated band intensities, not intensity per unit wavenumber. Full algorithmic details, parameter choices, and examples are provided in the Supporting Information (see Note S3).

### 4.6 Spectral Unmixing

In this work, we adopt the widely used Linear Mixing Model (LMM), in which each spectrum $x\in R^{b}$ is expressed as a linear combination of n endmember spectra $m_{i}\in R^{b}$:

(1) $$x=\sum_{i=1}^{n}\alpha_{i}m_{i}+\varepsilon$$

where $\alpha_{i}$ denotes the abundance of the i‐th endmember.

#### 4.6.1 Endmember Identification

To identify the endmember spectra from the data, we employed two well‐established blind unmixing algorithms (a)Vertex Component Analysis (VCA) [27], a fast geometrical method that projects the dataset along random directions to iteratively identify extreme points of the spectral simplex, which serve as candidate endmembers; and (2) *N‐FINDR* [43], an algorithm that searches for the set of endmembers maximizing the volume of the simplex formed by candidate spectra, under the assumption that pure pixels were present in the dataset. Both algorithms require specifying the number of endmembers in advance. Here, we constrained the problem to two endmembers (sp2 and sp3), which reflects the known physics of the system and avoids overfitting.

#### 4.6.2 Abundance Estimation

Once endmembers were identified, abundances could computed by solving the linear inverse problem under different constraints: (a) Fully Constrained Least Squares (FCLS) [28]: enforced both non‐negativity ($\alpha_{i}\geq 0$), and the sum‐to‐one constraint ($\sum_{i=1}^{n}\alpha_{i}=1$), ensuring abundances could be interpreted as fractions; and (b) Non‐Negative Least Squares (NNLS) [44]: relaxes the sum‐to‐one condition but maintains non‐negativity, which could be useful when baseline variations are present. As shown in Figure 3, the abundance maps at 50 μm s^−1^, the pristine diamond region did not show an abundance of exactly zero for the graphite endmember and one for the diamond endmember. This was expected since the endmembers were extracted across all wire maps, focus and intensity differences between datasets also shift the estimated simplex (see Note S8). The result was a small residual assignment of sp2 abundance even in pristine regions. Importantly, the maps still provide clear contrast: pristine areas were dominated by the sp3 endmember, while graphitized tracks were dominated by sp2, confirming the physical validity of the decomposition.

#### 4.6.3 Limits of Linear Mixing Model

Raman scattering was, to first order, linear in the number of scatterers, so spatial coexistence of sp3 and sp2 domains within the confocal volume leads to additive spectra, which the LMM captures. Moreover, our maps were recorded on polished surfaces with modest absorption at 532 nm outside the most heavily graphitized regions, reducing multiple scattering and re‐absorption that could induce non‐linear mixing. That said, three effects could challenge strict linearity: (i) strong broadband fluorescence in highly damaged zones, (ii) self‐absorption and re‐emission in thick, strongly absorbing tracks, and (iii) spectral distortions from stress/strain gradients that shift or broaden peaks in a way not representable by a fixed endmember. In such cases, non‐linear or bilinear models (e.g., polynomial post–nonlinear mixing, kernel‐based unmixing, or autoencoder‐based unmixing [45]) may yield incremental gains, at the cost of additional parameters and reduced interpretability.

#### 4.6.4 Implementation

We conducted the hyperspectral unmixing analysis with Python using the RamanSPy package [46].

## Conflicts of Interest

The authors declare no conflicts of interest.

## Supporting information


**Supporting File**: smtd70579‐sup‐0001‐SuppMat.pdf.

## Data Availability

The data that support the findings of this study are available from the corresponding authors, [X.C., A.F.G.], upon reasonable request.
