# Supplementary material for: Raman Microspectroscopy for Structural Indication in Ultrafast Laser Writing
Source: Small Methods. 2026 Mar 15;10(8):e02413. doi: 10.1002/smtd.202502413 (PMC13103637; doi:10.1002/smtd.202502413)
Supplement: Supplementary file 1 — Supporting File: smtd70579‐sup‐0001‐SuppMat.pdf. [file SMTD-10-e02413-s001.pdf]

Raman Microspectroscopy for Structural Indication in Ultrafast Laser Writing

Xingrui Cheng[1,*], Eugenio Picheo[2], Zhixin Chen[1,2],
Martin J. Booth[1], Patrick S. Salter[1], Álvaro Fernández–Galiana[1,*]

[1] Department of Engineering Science, University of Oxford, Parks Road, Oxford OX1 3PJ, UK
[2] Department of Materials, University of Oxford, Parks Road, Oxford OX1 3PH, UK

*Corresponding authors: xingrui.cheng@materials.ox.ac.uk, alvaro.fernandezgaliana@eng.ox.ac.uk

Supporting Information

Supporting Note 1: Pad optical micrograph and Raman map

As described in the manuscript, we employed laser-written graphitic pads and wires to emulate two common classes of conductive elements. Supporting Figure 1a shows an optical micrograph of pad electrodes with a width of 50 μm and length of 200 μm, written at scan speeds of 100 and 200 μm s^{-1}, respectively. The 100 μm s^{-1} pad exhibits a resistance of $214.X$ $\Omega \pm 10.4$ Ω, whereas the 200 μm s^{-1} pad shows $3,69X.XX$ $\Omega \pm 1.59$ Ω, i.e., a difference of more than an order of magnitude. Under optical illumination, the laser-written tracks appear dark relative to the surrounding single-crystal diamond. This contrast arises because converting transparent, wide-band-gap sp3 diamond to disordered sp2 carbon increases visible-wavelength absorption and slightly raises the refractive index, thereby reducing transmittance through the modified region [1, 2]. Supporting Figures 1b and c present the corresponding photoluminescence (PL) and Raman (sp3 and sp2) maps. In both pads, the written regions appear bright in PL immediately after fabrication. This behavior differs from the wires, which appear dark in PL due to laser-induced non-radiative recombination pathways.

The bright pad emission consists of locally activated NV^0 and NV$^-$ centers in this Type Ib diamond (high nitrogen content), and other defect centers that producing a broad band that photo-bleaches under green (532 nm) excitation. This highlights that monitoring the full-window PL signal is not an ideal metric during fabrication, as relevant phase information is obscured by unrelated emissions and does not directly reflect device performance. In contrast, hyperspectral Raman mapping provides more specific structural information. By integrating the sp3 diamond window (1325-1340cm^{-1}) [3] and the sp2 G-band window (1575-1610cm^{-1}) [1], the sp3 map shows the laser-written regions as decreased peak intensity relative to pristine diamond, with stronger depletion for the lower scan speed. The sp2 map exhibits the expected behavior (i.e., increased peak intensity in the written region) but has lower overall contrast. Both the sp3 and sp2 Raman maps exhibit horizontal line-like features, which arise from the writing process. Such features are more pronounced in the sp2 than in the sp3 maps. A detailed explanation is provided in Supporting Note 2.

Supporting Figure 2 shows an optical micrograph of a pad acquired after PL mapping. Scanning a >20 mW, 532 nm beam around the electrode removes the initial femtosecond-laser-induced graphite debris [2], revealing a clean structure (dashed green square) compared to the post-writing state (dashed red square). Thus, maintaining the 532 nm probe during Raman acquisition could serve the dual role of (a) enabling spectroscopy monitoring, and (b) clearing graphite debris.

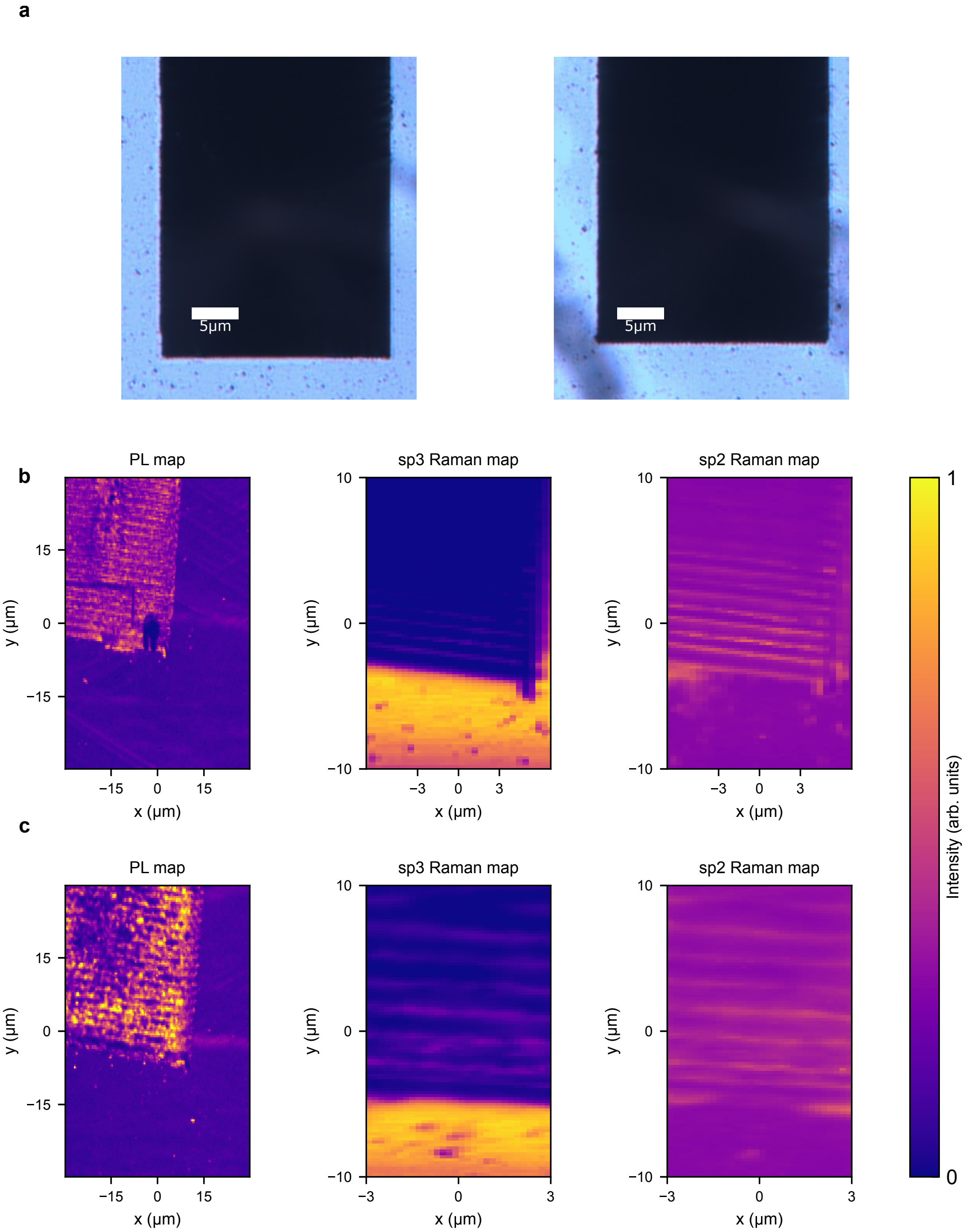

Supporting Figure 1: **Laser-written graphitic structures with optical/PL/Raman characterization.a-c)** Optical micrographs, PL maps, and Raman intensity maps (sp3 and sp2 windows) for *pads* written at scan speeds of 100 and 200 μm s^{-1}, respectively. The 100 μm s^{-1} pad exhibits a resistance of $214.X$ $\Omega \pm 10.4$ Ω, whereas the 200 μm s^{-1} pad exhibits $3,69X.XX$ $\Omega \pm 1.59$ Ω, i.e., a difference exceeding one order of magnitude. The optical darkening and PL contrast are effectively indistinguishable between the two speeds; thus, optical micrographs and PL maps cannot resolve electrodes that differ in resistance by more than an order of magnitude. By contrast, Raman mapping of the sp3 window provides a direct and more reliable measure of the degree of graphitization, offering phase-specific insight that complements the broadband PL response and the lower-contrast sp2 map.

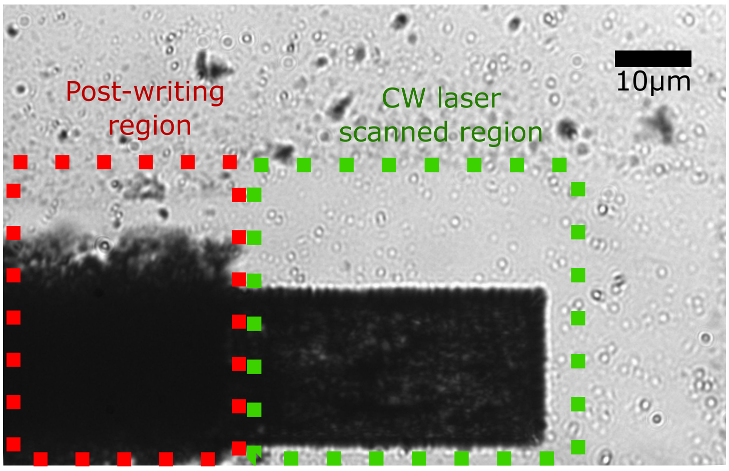

Supporting Figure 2: **CW-laser-assisted removal of graphitic debris from laser-written pads.** Optical micrograph of a graphitic pad after femtosecond (fs) laser writing. The dashed red box highlights debris generated during fs writing; the dashed green box marks a region subsequently scanned with a CW laser, where the surface graphitic debris has been effectively removed, revealing the underlying written structure.

Supporting Note 2: Origin of contrast in hyperspectral maps

As shown in Supporting Figure 3, both the sp3 and sp2 Raman maps exhibit horizontal line-like features, which are more pronounced in the sp2 map than in the sp3 map. The origin of these contrasts differs for the two cases. Since the pad is fabricated by stacking parallel wires with a 0.5 μm pitch, the bright features in the sp3 maps correspond to regions between adjacent wires where less damage occurs, resulting in reduced graphitization. In contrast, the bright features in the sp2 maps arise from a different mechanism, consistent with the behavior described in Figure 1c of the main text. Here, the increased brightness is not related to enhanced graphitization but instead originates from broadband background emission.

To confirm this, Supporting Figure 3 presents a Raman map in the 590–600 nm spectral range (the same hyperspectral map of Figure 1c in the main text but different spectral window), excluding the sp3 and sp2 bands. Compared to the laser-written wire and the pure diamond background, the surrounding damaged shell appears bright, confirming that the observed contrast does not originate from either the sp3 or sp2 Raman signals. Spectra from three representative pixels—assigned to the graphite core, the damaged shell, and the pure diamond background—are extracted in Supporting Figure 3a and shown in Supporting Figure 3b–d. Each spectrum is normalized for comparison. The graphite region exhibits depleted sp3, a clear D band, and sp2 features, while the pure diamond spectrum is dominated by the sp3 line. In the damaged shell, no D or sp2 bands are observed apart from the sp3 peak. Instead, a broad emission is present, which contributes to the increased brightness observed in both the photoluminescence and hyperspectral maps. This emission is attributed to defect activation in the damaged shell surrounding the wire core [3].

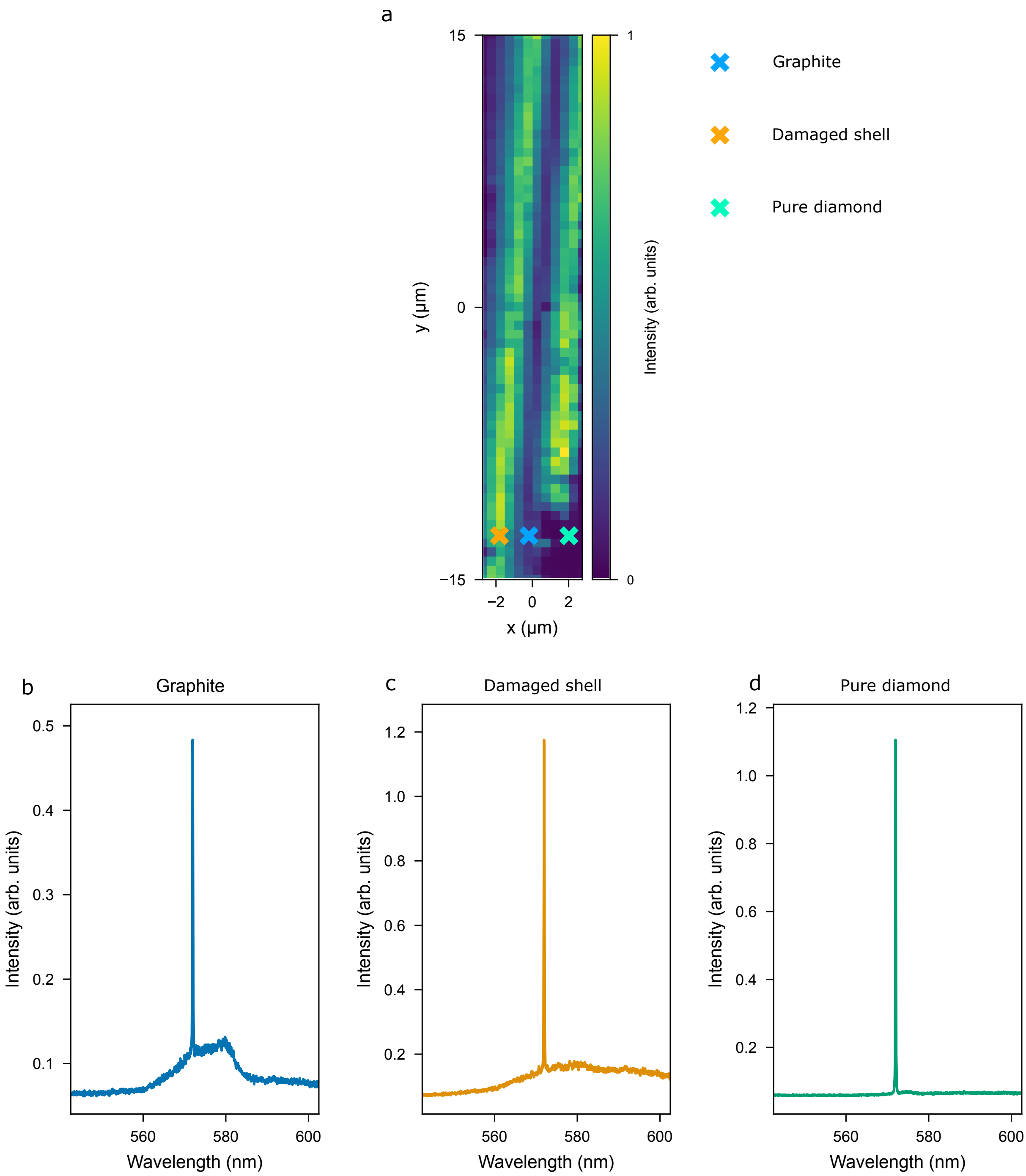

Supporting Figure 3: **Raman hyperspectral analysis of a laser-written wire with surrounding damaged shell. a)** Raman map in the 590–600 nm range, excluding sp3 and sp2 bands. Bright regions surrounding the wire correspond to broadband background emission. Colored crosses mark representative pixels: blue for the graphite core, orange for the damaged shell, and light green for the pure diamond background. **b–d)** Self-normalized spectra from the selected pixels. The graphite spectrum (blue) shows depleted sp3, D, and sp2 features; the diamond spectrum (light green) is dominated by the sp3 line; while the damaged shell (orange) lacks D and sp2 bands but exhibits broad background emission attributed to defect activation.

Supporting Note 3: Pixel identification, integration, and normalization

The hyperspectral Raman data were loaded as a three-dimensional array $I(x, y, \lambda)$, where x and y represent the spatial coordinates, and λ represents the wavelength. To compensate for experimental variations including focusing, and excitation laser power fluctuations between different datasets, we implemented a sample-specific normalization procedure based on pristine diamond regions (shown as green pixels in Fig. 4).

For the sp3 diamond peak analysis, we defined a wavelength window $\Lambda_{sp3} = [1325, 1340]$ cm^{-1}. The integrated intensity for each pixel (i, j) was calculated as:

$$I_{int}(i,j) = \sum_{\lambda \in \Lambda_{sp3}} I(i,j,\lambda) \tag{1}$$

For every sample, we identified the N_{bright} brightest pixels (where $N_{bright} = 200$ for wire electrodes and $N_{bright} = 1000$ for pad electrodes) by sorting all pixels according to their integrated intensity values. These brightest pixels correspond to pristine diamond regions that serve as our normalization baseline (see Fig. 4 where the green pixels indicate the normalization baseline). The normalization factor was computed as the mean intensity of these brightest pixels:

$$F_{norm} = \frac{1}{N_{bright}} \sum_{k=1}^{N_{bright}} I_{int}(p_k) \tag{2}$$

where p_k represents the k-th brightest pixel.

After establishing the normalization factor, the entire hyperspectral image was normalized:

$$I_{norm}(i,j,\lambda) = \frac{I(i,j,\lambda)}{F_{norm}} \tag{3}$$

Subsequently, we extracted and performed integrated intensity calculations for both the sp3 (diamond) and sp2 (graphitic carbon) spectral windows for those with dark sp3 intensity (red pixels in Fig. 4). For each pixel, we computed:

$$I_{sp3}^{norm}(i,j) = \sum_{\lambda \in [1325,1340]} I_{norm}(i,j,\lambda) \tag{4}$$

$$I_{sp2}^{norm}(i,j) = \sum_{\lambda \in [1575,1610]} I_{norm}(i,j,\lambda) \tag{5}$$

From the normalized sp3 integrated intensity map, we identified the N_{dark} darkest pixels (typically 60-2000 pixels depending on the sample types), which correspond to areas with reduced diamond features, indicating graphitization or structural modification.

The normalization procedure establishes a reference scale where a value of 1.0 represents the average integrated intensity of pristine diamond in the sp3 window (1325-1340 cm^{-1}). Mathematically, $\langle I_{sp3}^{norm} \rangle_{pristine} = 1.0$. This normalization enables direct comparison between different datasets and quantitative assessment of the depletion of sp3 Raman signal. For the darkest pixels identified in our analysis, $I_{sp3}^{norm} < 1.0$ indicates reduced diamond sp3 character relative to pristine regions, while I_{sp2}^{norm} values can be directly compared to the sp3 baseline. The ratio $R = I_{sp2}^{norm}/I_{sp3}^{norm}$ provides a quantitative metric for the degree of graphitization, where $R \ll 1$ indicates predominantly diamond sp3 character and $R \geq 1$ suggests substantial sp2 carbon content.

For each identified dark pixel, we exported the normalized integrated intensities for both spectral windows along with their spatial coordinates. The average normalized intensity across all dark pixels was calculated as:

$$\bar{I}_{window}^{dark} = \frac{1}{N_{dark}} \sum_{k=1}^{N_{dark}} I_{window}^{norm}(d_k) \tag{6}$$

where d_k represents the k-th darkest pixel and $window \in \{sp3, sp2\}$. This averaging procedure provides a statistical measure of the overall modification in the analyzed region, with the normalized scale ensuring that values can be directly compared across different measurements and sample positions. The exported data enables quantitative comparison of the degree of graphitization across different electrode

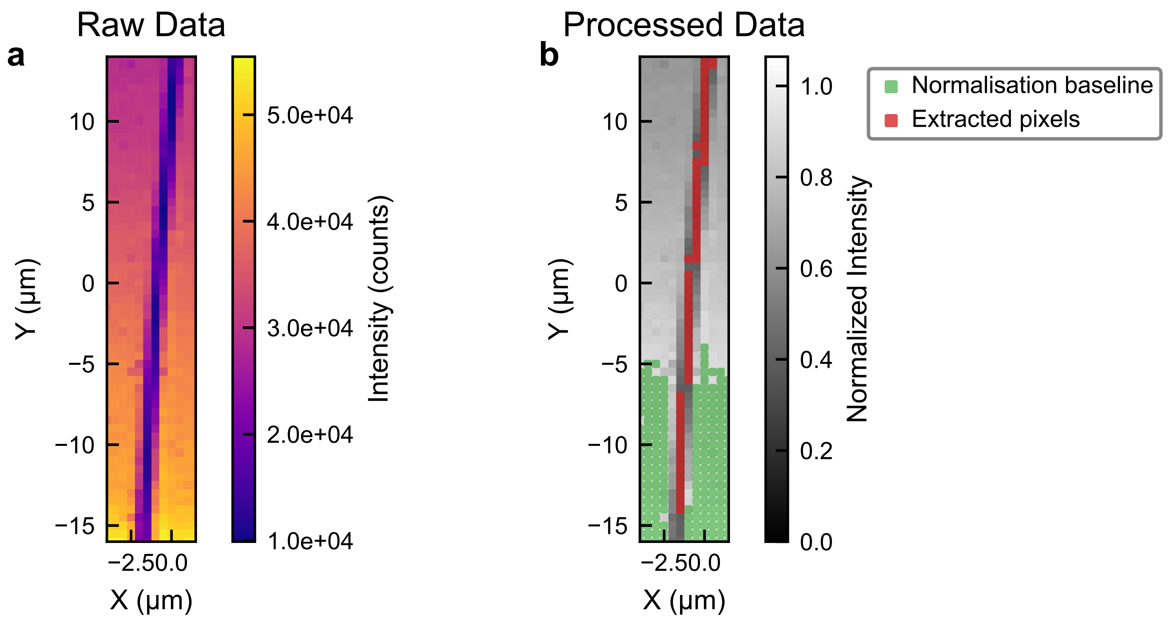

Supporting Figure 4: **Normalization and pixel selection for hyperspectral Raman analysis. a**, Raw Raman map integrated over the sp3 window $[1325, 1340]$ cm^{-1}. **b**, Processed map after normalization to pristine diamond. Green pixels indicate the N_{bright} highest sp3-integrated intensities used to compute the normalization factor F_{norm} (baseline), and red pixels indicate the N_{dark} selected low-sp3 locations used for further analysis. The grayscale reports normalized sp3 intensity with the pristine-diamond baseline satisfying $\langle I_{sp3}^{norm} \rangle = 1.0$. This procedure enables direct comparison of I_{sp3}^{norm}, I_{sp2}^{norm}, and the ratio $R = I_{sp2}^{norm}/I_{sp3}^{norm}$ across datasets. Axes are X-Y in μm.

configurations and processing conditions. We note that the sp2 integration window (15 cm^{-1}) is wider than the sp3 window (35 cm^{-1}), reflecting the intrinsic spectral widths of these features in diamond. As all measurements use identical integration windows, this systematic difference does not affect relative comparisons between samples. The sp2/sp3 ratio should therefore be interpreted as the ratio of integrated intensities over their respective characteristic spectral features, rather than intensity per unit wavelength.

Supporting Note 4: Quantitative PL analysis

To evaluate whether broadband photoluminescence (PL) intensity can serve as an in situ process indicator, we extracted the PL intensity from the written pixels. The results are presented in Supporting Figure 5. This figure shows a correlation between the resistance and the normalized PL intensity of the written wire electrodes, which suggests that broadband PL intensity can be leveraged to assess performance of the written electrodes.

Supporting Figure 5 also compares the PL with the sp3 intensity metric proposed in the main manuscript. This comparison showcases how the normalized PL follows the sp3 trend: PL increases with resistance, consistent with a larger fraction of diamond-like bonding. Yet, despite having similar correlation with the resistance of the electrodes, the PL normalized intensity presents significantly larger variance in the measurements. This is due to the broad spectral collection window of these PL maps ($\sim$550-850 nm), which can include emission from phenomena not directly related to graphitization (i.e., in Type Ib diamond intrinsic defects generate a non-negligible background in PL maps).

Thus, even if broadband PL can serve as a quantitative indicator during laser fabrication, in practice it is less specific and less accurate than the sp3 intensity, which should be favored for in situ process monitoring.

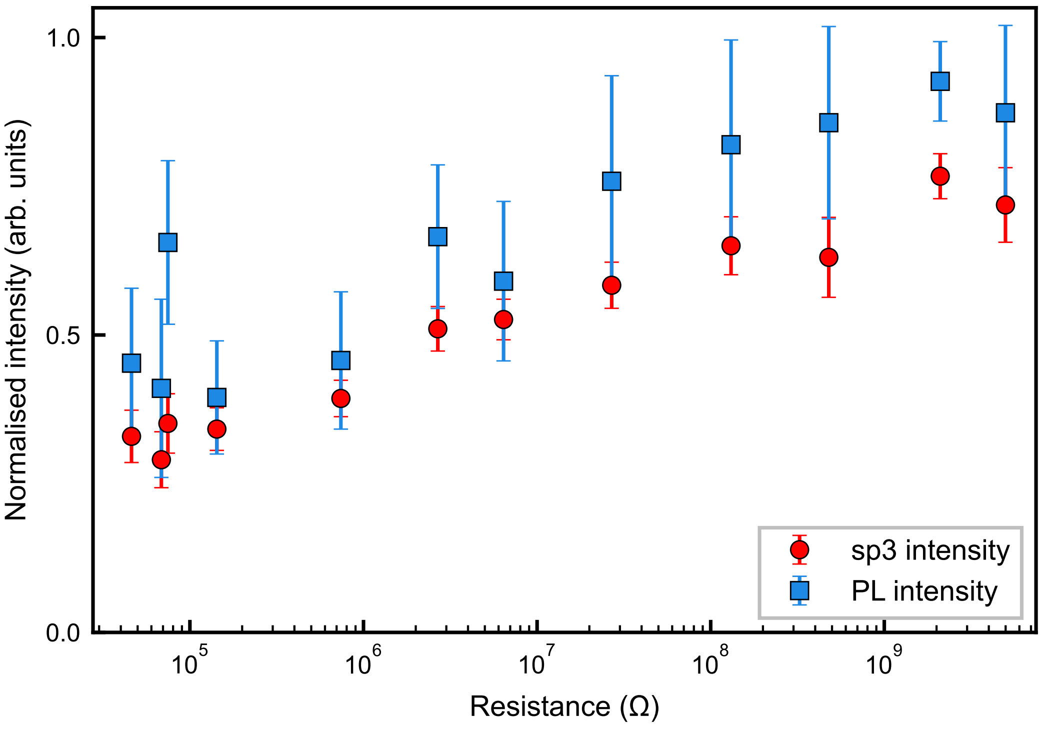

Supporting Figure 5: **Broadband PL intensities analysis for wire electrodes.** Normalized broadband PL intensity (blue squares) and sp3 Raman intensity (red circles) plotted versus electrode resistance for laser-written wires at varying scan speeds. PL was collected for a spectral window of 550-850 nm and both signals were normalized using high-sp3 ('pure-diamond') reference pixels (see Supporting Note 3). Error bars indicate 1σ.

Supporting Note 5: sp3 distribution

The speed-dependent probability-density histograms of the normalized sp3 Raman intensity, computed from pixels identified as graphitized in the hyperspectral maps, are shown for pad and wire electrodes in Supporting Figure 6 and Supporting Figure 7, respectively. For each scan speed, 1500 pixels are analyzed for pads and 60 for wires. Intensities are referenced to pristine diamond, so higher normalized sp3 corresponds to weaker graphitization.

In pad electrodes, increasing scan speed shifts the distributions toward higher values and simultaneously broadens them, consistent with reduced energy deposition per unit length and a less uniform sp3-to-sp2 transformation. By contrast, wire electrodes exhibit narrower distributions across all scan speeds because their geometry prevents overwriting. Nonetheless, they display the same overall trend as pads: increasing scan speed shifts the distributions to higher normalized sp3 values, reflecting a lower degree of graphitization in the laser-written structures.

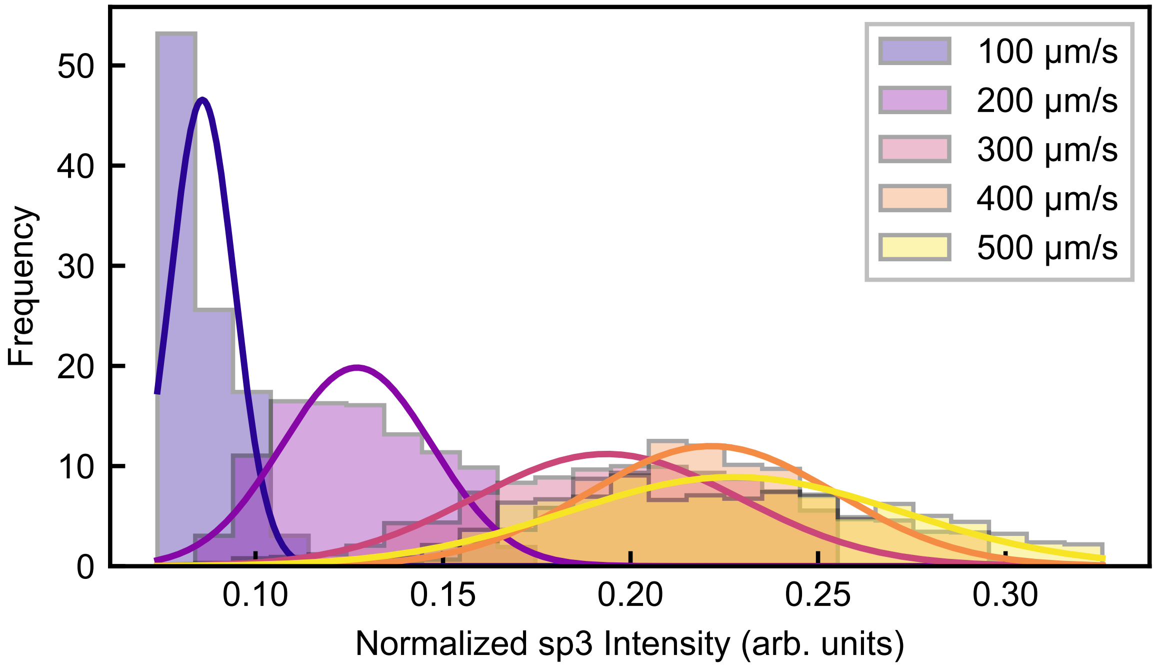

Supporting Figure 6: **Distribution of normalized sp3 intensity for pad electrodes at different scan speeds.** Probability-density histograms with normal distribution fits of the normalized sp3 Raman intensity from pads written at different scan speeds (1500 spectra per laser scan speed). Increasing scan speed shifts the distribution to higher sp3 intensity and broadens it, indicating reduced graphitization and increased phase inhomogeneity.

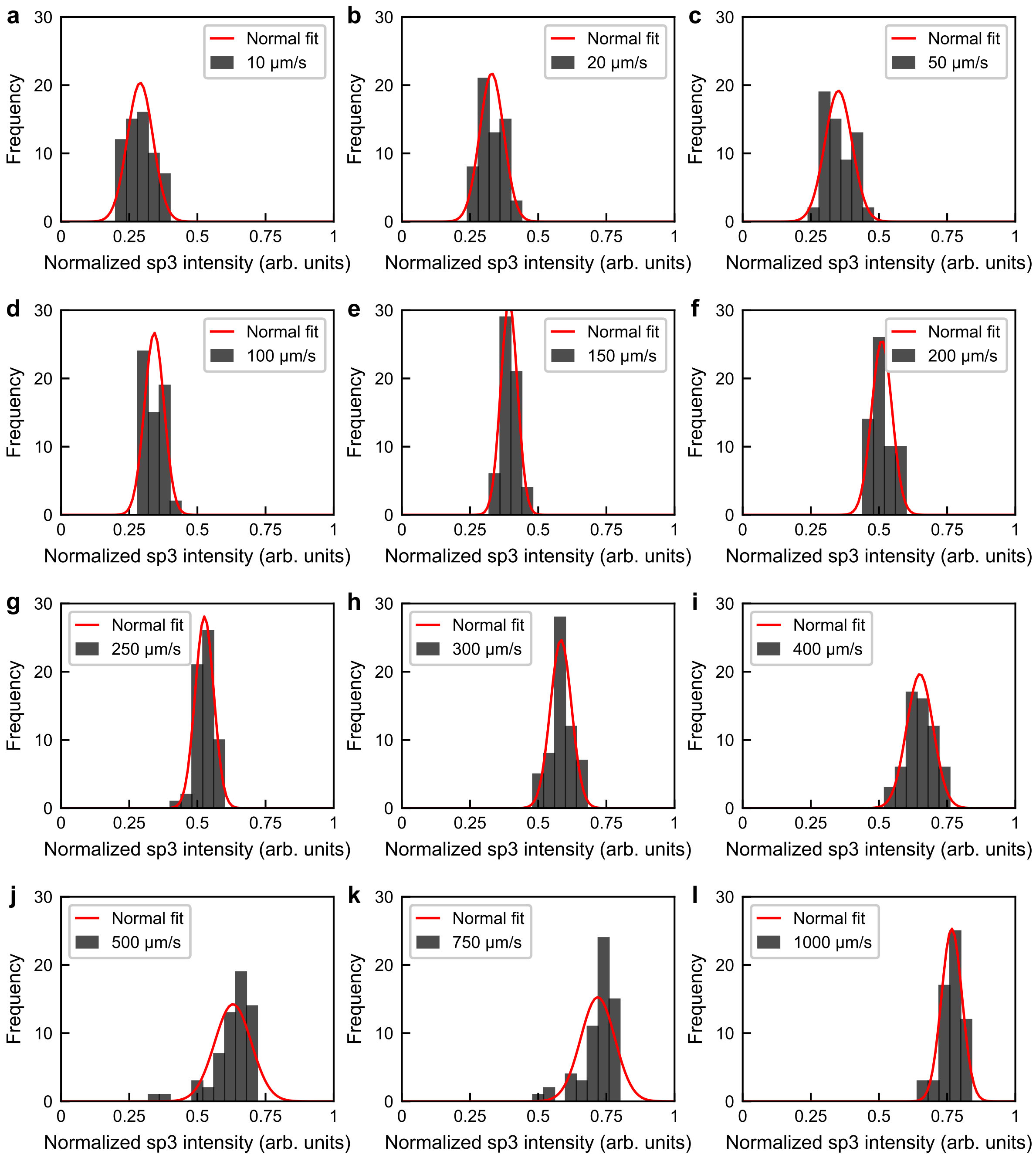

Supporting Figure 7: **Distribution of normalized sp3 intensity for wire electrodes at different scan speeds.** Histograms of 60 pixels per electrode with corresponding normal distribution fits are shown for scan speeds ranging from 10 μm s^{-1} to 1000 μm s^{-1}. The absence of overwriting in wire electrodes results in narrower distributions compared to pad electrodes. Increasing scan speed shifts the distributions toward higher sp3 values, consistent with reduced deposited energy per unit length and a lower degree of graphitization.

Supporting Note 6: sp3 Raman analysis

To complement the two-probe transport data, we quantified how the sp3 Raman response varies with laser scan speed. For each speed, 60 pixels within the graphitized region of a laser-written wire were averaged and the sp3 peak was fitted with a Lorentzian. Averaged spectra for scan speeds from 10 to 1270 μm s^{-1} were analyzed; the resulting peak center and full width at half maximum (FWHM) are plotted in Fig. 8 as blue circles and dark-orange squares, respectively. A pristine-diamond reference,

obtained by averaging 100 pixels from an unexposed area, is shown at 3000μm s^{-1} for visual comparison only.

Across the series, the sp3 mode lies slightly above 1332cm^{-1}, consistent with tensile stress in the transformed graphite layer and the concomitant in-plane compressive stress imparted to the adjacent diamond[2], which produces a blue shift of the diamond Raman mode [4, 5]. The FWHM decreases systematically with increasing scan speed. Slower scans deposit more energy per unit length, driving a higher degree of graphitization; accordingly, the sp3 line departs from an ideal Lorentzian, broadening and sometimes becoming asymmetric. This behavior reflects increasing lattice disorder and strain distributions (inhomogeneous broadening), reduced phonon lifetimes (homogeneous broadening), and phonon-confinement effects; if the material becomes sufficiently conducting, Fano interference with an electronic continuum can further skew the lineshape [6, 7]. All averaged spectra and their Lorentzian fits are provided in Fig. 9.

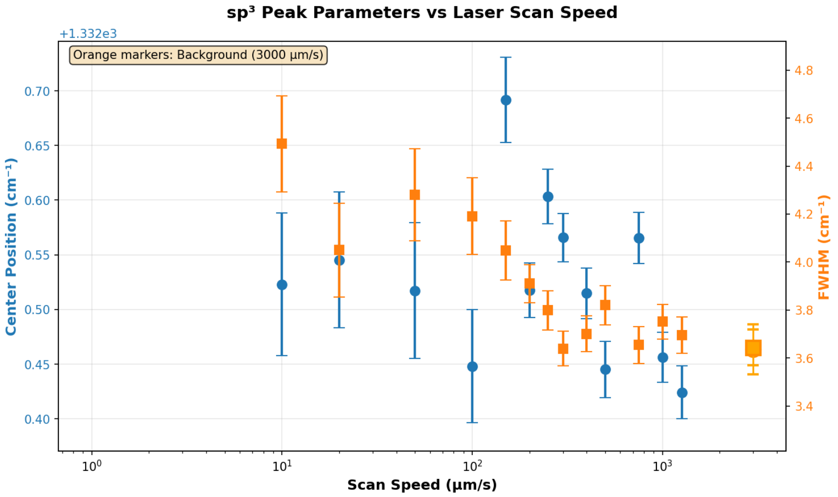

Supporting Figure 8: **sp3 Raman peak evolution with laser scan speed in wire electrodes.** Mean center position (blue circles, left axis) and FWHM (dark-orange squares, right axis) extracted from Lorentzian fits to averages of 60 pixels within the graphitized wire region for each speed (10-1270 μm s^{-1}). A pristine-diamond reference (100-pixel average from an unexposed area) is placed at 3000 μm s^{-1} for visual comparison. The center exhibits a slight blue shift relative to 1332 cm^{-1}, consistent with stress coupling between the graphitized layer and the surrounding diamond, while the FWHM narrows with increasing scan speed, indicating reduced disorder at higher speeds. Error bars indicate pixel-to-pixel variability.

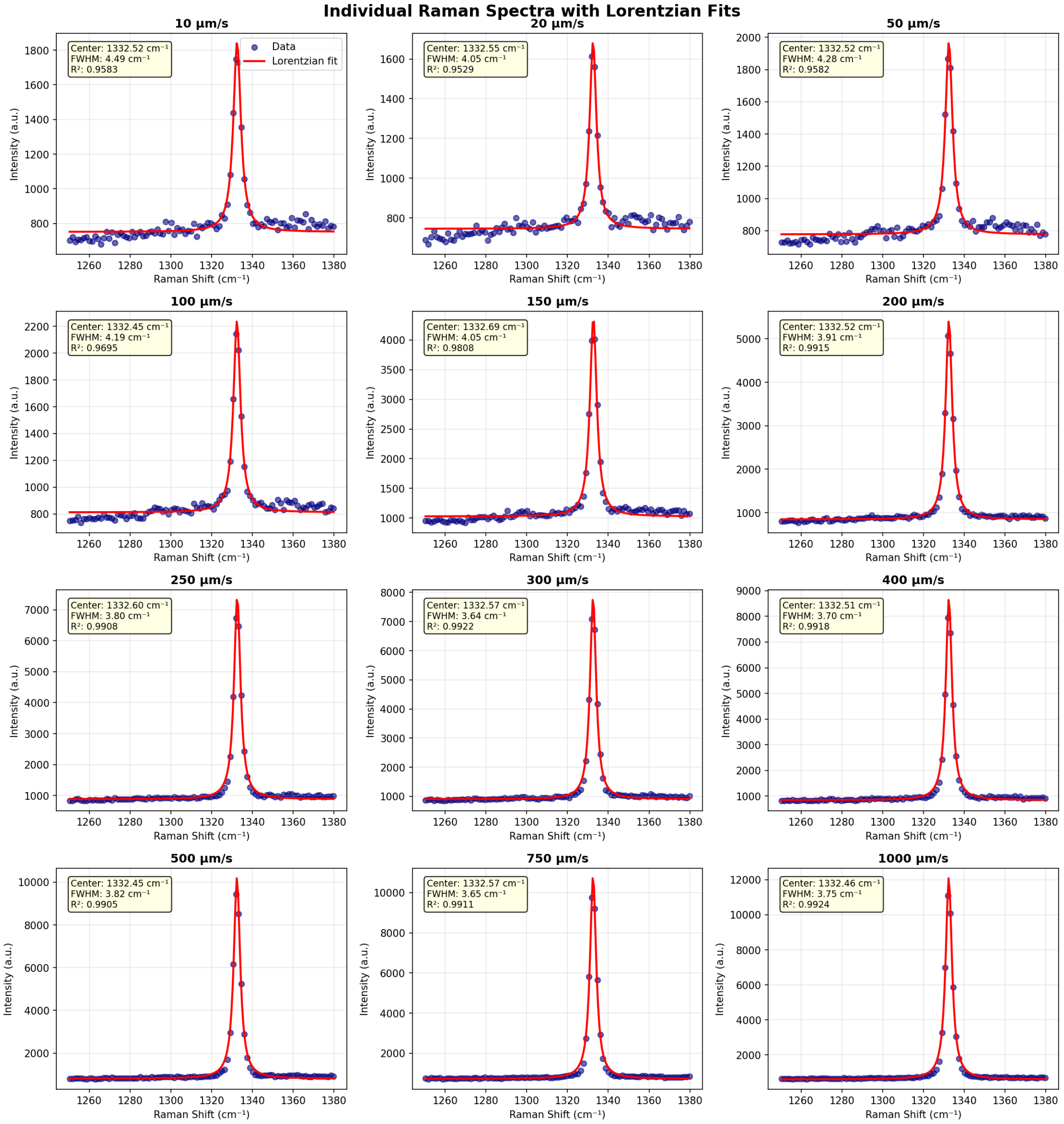

Supporting Figure 9: **Representative sp3 Raman spectra and Lorentzian fits at different scan speeds.** Averaged spectra from 60 pixels within the graphitized wire region are shown for laser scan speeds ranging from 10 to 1000 μm s^{-1}. Data are plotted as blue points and Lorentzian fits as red curves. The extracted peak center position, FWHM, and coefficient of determination (R^2) for each fit are indicated in the insets. At slower scan speeds, the sp3 mode broadens significantly, reflecting enhanced graphitization, lattice disorder, and strain distributions, while higher scan speeds produce narrower peaks, indicative of reduced disorder and improved crystalline retention.

Supporting Note 7: Electrical performance for both pad and wire

Electrical measurements confirm that femtosecond-laser writing produces ohmic graphitic pathways whose conductance scales with scan speed and tracks the depletion of sp3 Raman intensity in the written region. For wire electrodes, the I-V characteristics are strictly linear from -0.5 to +0.5 V, and linear fits yield resistances that vary over several orders of magnitude as the scan speed increases from 10 to 1000 μm s^{-1} (Fig. 10a-l). At the slowest scans (10-50 μm s^{-1}) the wires exhibit resistances in the tens of kΩ, approaching the series resistance floor of the test board contact pads, whereas progressively faster scans—depositing less energy per unit length—produce markedly higher resistances reaching the MΩ-GΩ

range.

Pad electrodes follow the same trend but with substantially lower absolute resistance due to their larger cross-section and multi-track overwriting, which yields more complete graphitization as indicated by the depleted sp3 Raman signal. I-V curves acquired from -0.1 to +0.1 V remain perfectly linear, with fitted resistances rising from a few hundred Ω at 100 μm s^{-1} to ∼50k Ω at 500 μm s^{-1} (Fig. 11a-e). The comparison between wires and pads highlights the expected geometric scaling and the writing process: wider, thicker pads support significantly higher conductance than narrow wires under otherwise identical conditions, and the zig-zag writing strategy introduces controlled overwriting that further enhances graphitization.

**Writing speed**	**Pad**		**Wire**	
	**Resistance**	**Uncertainty**	**Resistance**	**Uncertainty**
10 μm/s	-	-	68.366 kΩ	±0.011 kΩ
20 μm/s	-	-	46.186 kΩ	±0.014 kΩ
50 μm/s	-	-	74.764 kΩ	±0.009 kΩ
100 μm/s	214.08 Ω	±0.10 Ω	142.621 kΩ	±0.015 kΩ
150 μm/s	-	-	741.447 kΩ	±0.029 kΩ
200 μm/s	3.6951 kΩ	±0.0016 kΩ	2.68323 MΩ	±0.00010 MΩ
250 μm/s	-	-	6.41152 MΩ	±0.00022 MΩ
300 μm/s	24.270 kΩ	±0.003 kΩ	26.8711 MΩ	±0.0025 MΩ
400 μm/s	45.822 kΩ	±0.006 kΩ	130.589 MΩ	±0.021 MΩ
500 μm/s	53.545 kΩ	±0.008 kΩ	482.65 MΩ	±0.05 MΩ
750 μm/s	-	-	4830 MΩ	±30 MΩ
1000 μm/s	-	-	2110.8 MΩ	±0.7 MΩ

Supporting Table 1: Measured resistance for pads and wires at different writing speeds.

The electrical evolution with scan speed mirrors the Raman signatures of the laser-induced carbon. In both geometries, depletion of sp3 intensity and the concurrent strengthening of sp2 features at slower scan speeds coincide with reduced resistance, whereas the relative persistence of sp3 character at faster scans coincides with increased resistance. This direct Raman-electrical correlation substantiates that efficient graphitization—conversion from sp3 to sp2 bonding—governs charge transport in the written structures.

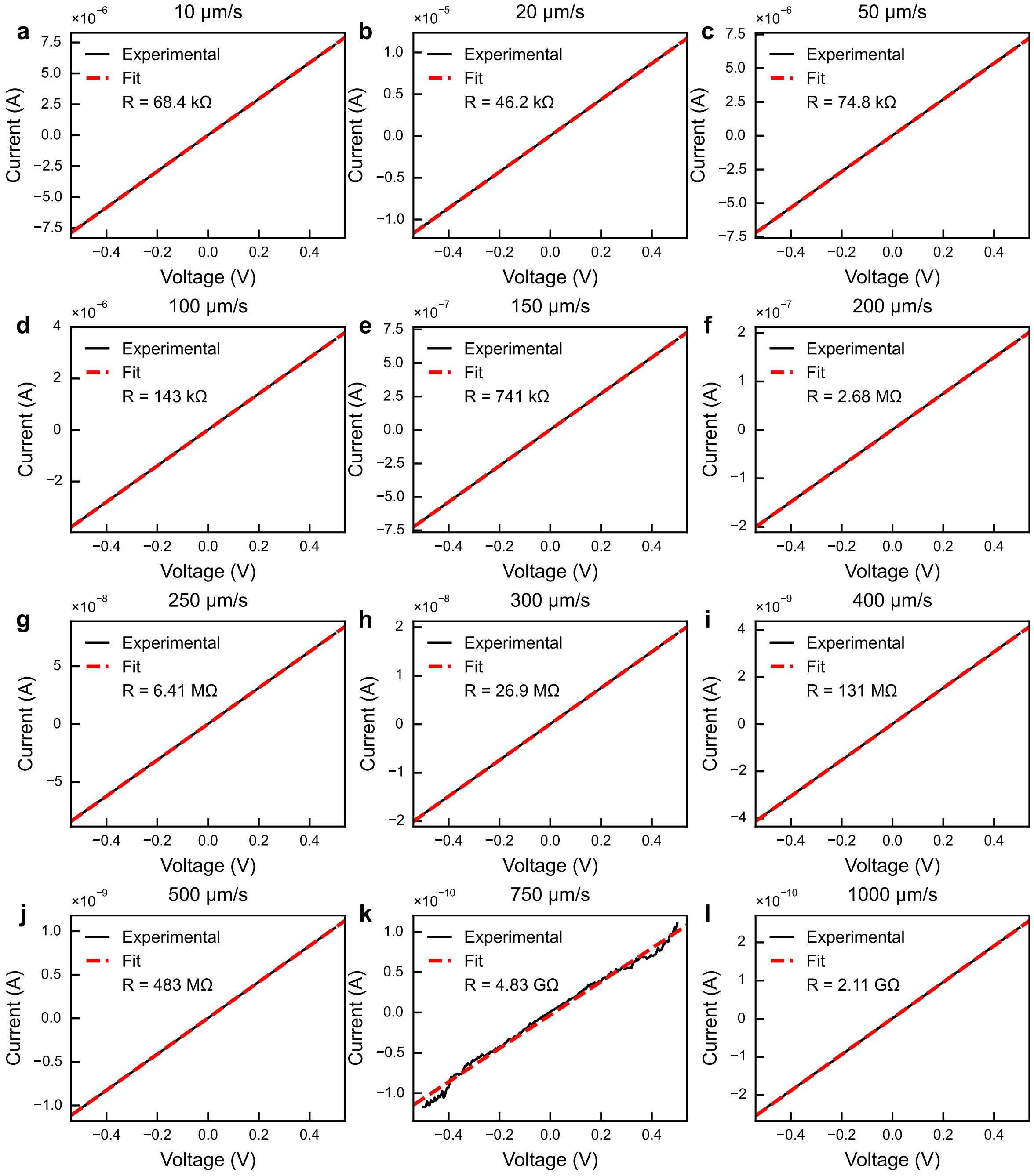

Supporting Figure 10: **Scan-speed dependent transport in laser-written wire electrodes. a-l**, Current-voltage characteristics (black) with linear fits (red, dashed) for wires written at 10, 20, 50, 100, 150, 200, 250, 300, 400, 500, 750, and 1000 μm s^{-1}, measured over -0.5 to $+0.5$ V. All traces are ohmic and the fitted resistances increase by orders of magnitude as the scan speed rises, evolving from the kΩ regime at slow scans to the MΩ-GΩ range at fast scans. The reduction in resistance at slow scans correlates with the depletion of sp3 Raman intensity and the concurrent strengthening of sp2 signatures, evidencing progressive graphitization.

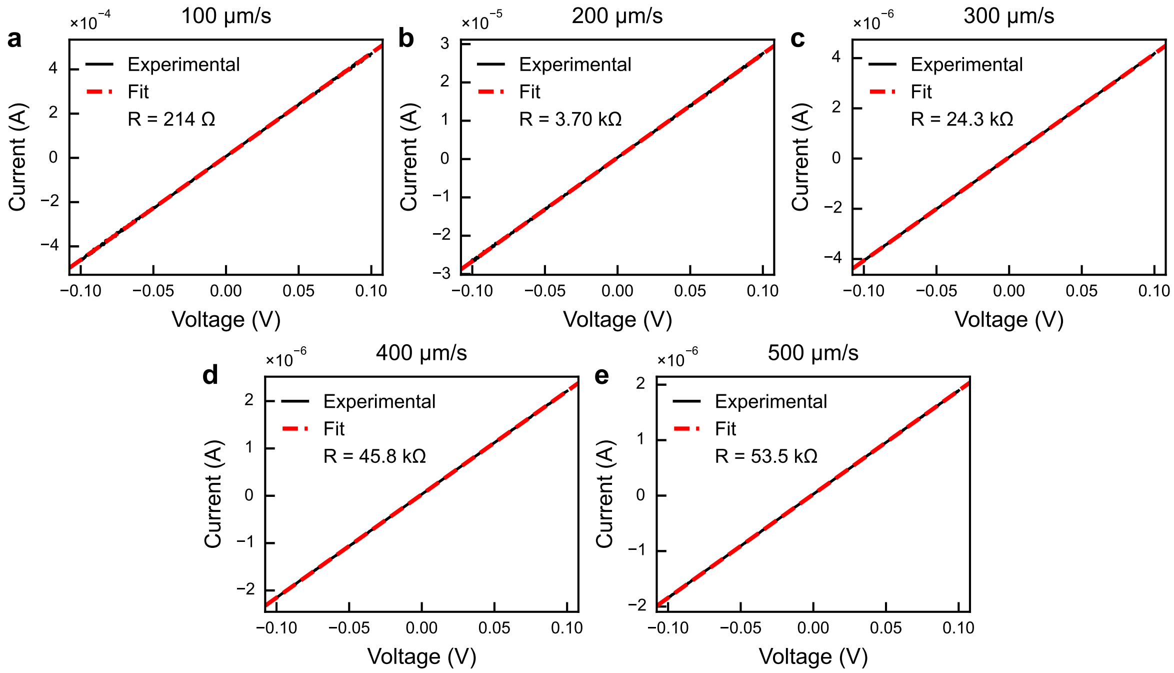

Supporting Figure 11: **Pad electrodes: lower absolute resistance with the same scan-speed trend. a-e)** IV characteristics (black) and linear fits (red, dashed) for pads written at 100, 200, 300, 400, and 500 μm s^{-1}, measured over -0.1 to $+0.1$ V. The response is strictly ohmic, with fitted resistances rising from $\sim$214 Ω at 100 μm s^{-1} to $\sim$53.5 kΩ at 500 μm s^{-1}. Compared with wires, pads exhibit substantially lower absolute resistance due to their larger cross-section and multi-track overlap. As in the wires, the decrease in resistance at slower scans tracks the depletion of sp3 intensity and the growth of sp2 features in the Raman spectra, confirming a direct Raman-electrical correlation.

Supporting Note 8: Hyperspectral unmixing

We performed hyperspectral unmixing with a linear mixing model using vertex component analysis (VCA) [8] for endmember extraction and fully-constrained least squares (FCLS) for abundance estimation. As shown in Supporting Figure 12, the unmixing results consistently show progressive loss of the diamond-associated component with decreasing scan speed, while the carbon-associated component increases and eventually saturates. This reinforces the main conclusion that sp^3 depletion provides a reliable, monotonic indicator of graphitization. Hyperspectral unmixing is a powerful technique that leverages the full spectrum and does not require pre-defined labels. However, it is sensitive to the presence of noise or species not considered in the analysis. As shown in Supporting Figure 12a, the presence of the broadband emission damage shell when fabricating at intermediate speeds is mislabeled as being a mixture of diamond and graphite phases, since in the analysis only two endmembers are considered.

Supporting Figure 13 presents the endmembers extracted using different unmixing techniques and spectral regions of interest (ROI), exhibiting the variance that these endmembers can have. This behavior is expected: VCA and *N-FINDR* [9] are geometric algorithms that approximate the data as lying within a convex simplex. They perform best when pixels lie close to the true extremes, but in practice the confocal voxel contains mixtures, variable SNR, NV^0 fluorescence, and potentially stress-induced peak shifts. The algorithms therefore identify different "extreme" representatives of the diamond-like and graphite-like responses, leading to small method-dependent variations that do not alter the physical interpretation. Indeed, the endmembers preserve the spectral features of their associated spectra, that is, the sp3 peak and NV0 band for the endmember associated to diamond, and the G band and, to lesser extent, the D band for the one associated to graphite.

Notably, for the spectral ROI including the sp3 peak, the large dynamic range of this feature amplifies small fluctuations which can distort the convex geometry and bias endmember selection. Excluding this line (spectral ROI: 1345–1800 cm^{-1}) reduces leverage and produces noisier but qualitatively similar maps.

Supporting Figure 13 also provides the first two principal components. It is important to distinguish

geometric unmixing from principal component analysis (PCA) [10]. VCA and N-FINDR seek spectra at the boundaries of the data cloud that can be interpreted as approximate endmembers, whereas PCA decomposes variance into orthogonal components that need not resemble real spectra and can take negative values. PCA is valuable for denoising and diagnostics, but its components cannot be interpreted as abundances.

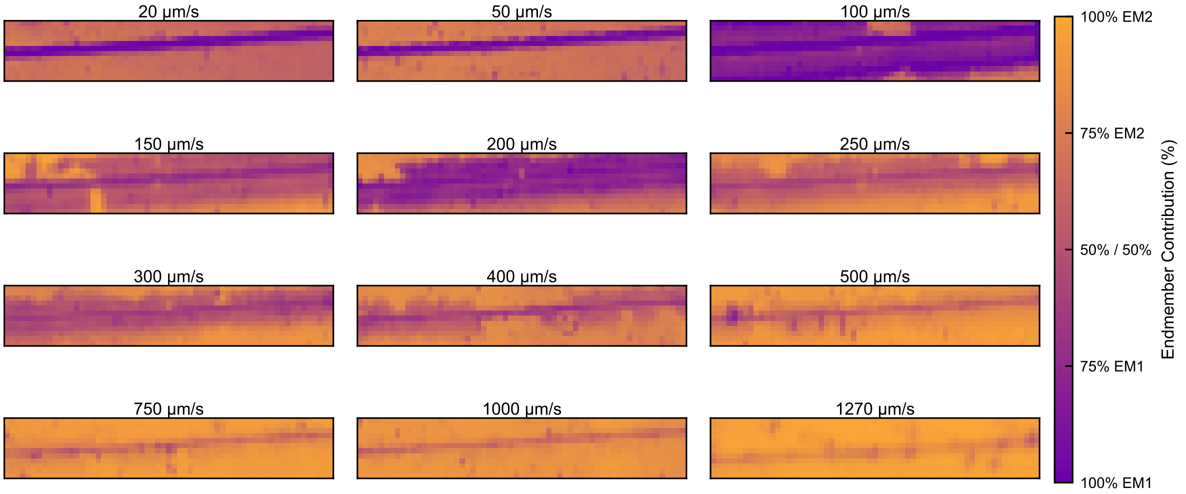

(a) Spectral unmixing (VCA) on all wires.

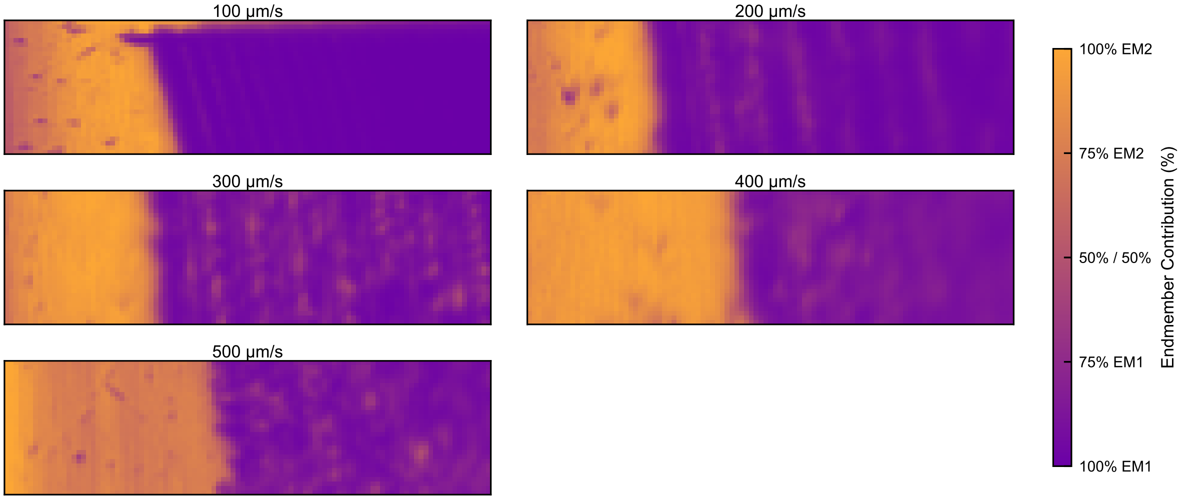

(b) Spectral unmixing (VCA) on all pads.

Supporting Figure 12: **Spectral unmixing (VCA) on all wires and pads.** These abundances maps are obtained applying vertex component analysis unmixing with fully-constraint linear least squares endmember determination to each complete dataset independently. The specific endmembers can be found in Supporting Figure 12.

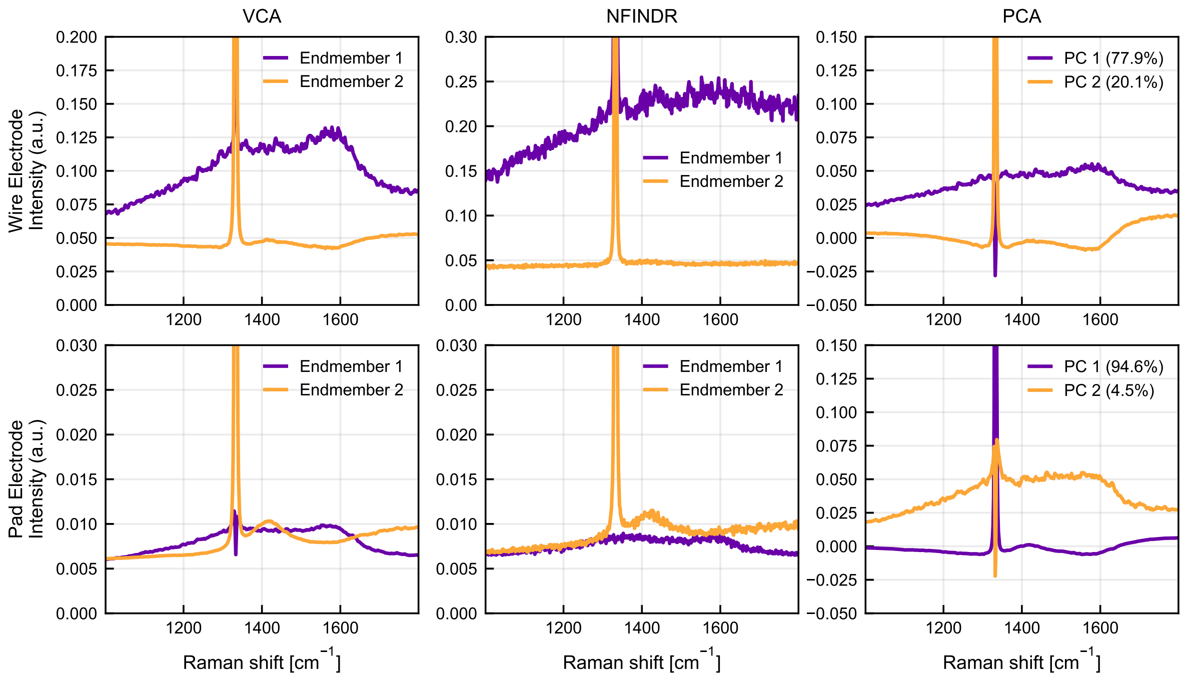

(a) Endmembers when unmixing in the spectral ROI including sp3 (1000-1800 cm^{-1}).

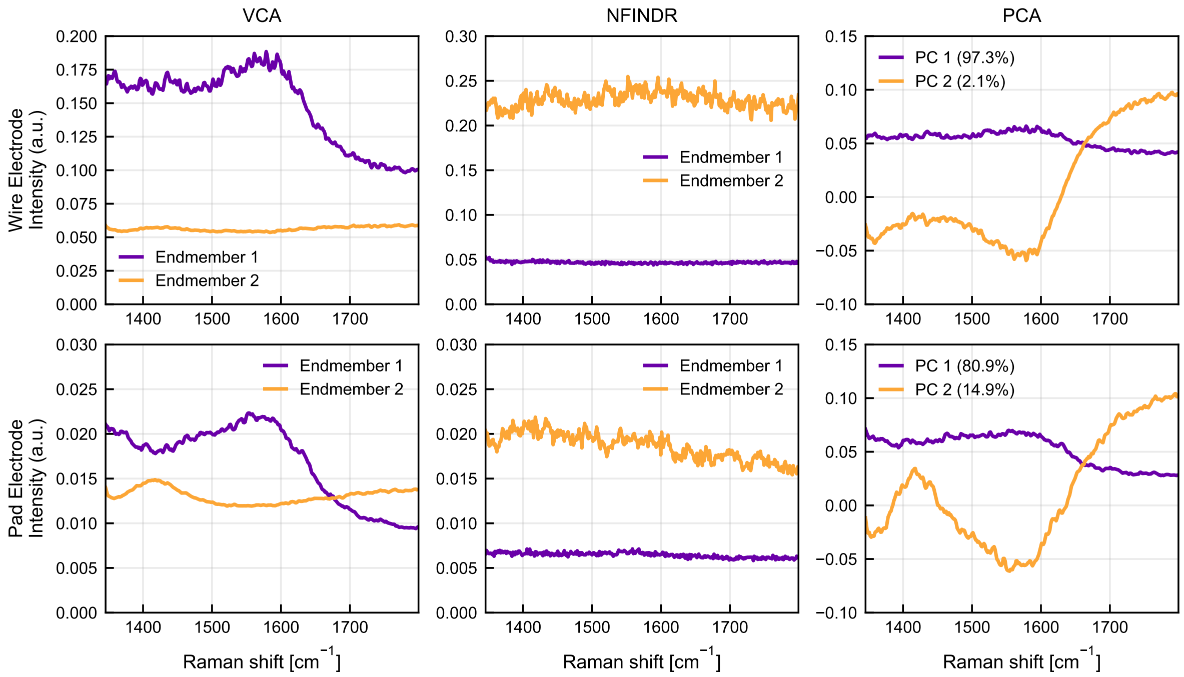

(b) Endmembers when unmixing in the spectral ROI not including sp3 (1345-1800 cm^{-1}).

Supporting Figure 13: **Endmembers for pad and wire electrodes.** Endmembers for the wire and pad electrodes using different unmixing methods. Also, the first two principal components resulting from principal component analysis, with their respective explained variance.

References

[1] Mark A Prelas, Galina Popovici, and Louis K Bigelow. *Handbook of industrial diamonds and diamond films*. CRC Press, 1997.

[2] C. Z. Wang, K. M. Ho, M. D. Shirk, and P. A. Molian. Laser-induced graphitization on a diamond (111) surface. *Phys. Rev. Lett.*, 85:4092–4095, Nov 2000.

[3] T. V. Kononenko, M. Meier, M. S. Komlenok, S. M. Pimenov, V. Romano, V. P. Pashinin, and V. I. Konov. Microstructuring of diamond bulk by ir femtosecond laser pulses. *Applied Physics A: Materials Science and Processing*, 90(4):645–651, March 2008.

[4] M. H. Grimsditch and A. K. Ramdas. Brillouin scattering in diamond. *Phys. Rev. B*, 11:3139–3148, Apr 1975.

[5] Alexander M Zaitsev. *Optical properties of diamond: a data handbook*. Springer Science & Business Media, 2013.

[6] J. O. Orwa, K. W. Nugent, D. N. Jamieson, and S. Prawer. Raman investigation of damage caused by deep ion implantation in diamond. *Phys. Rev. B*, 62:5461–5472, Sep 2000.

[7] S. Osswald, V. N. Mochalin, M. Havel, G. Yushin, and Y. Gogotsi. Phonon confinement effects in the raman spectrum of nanodiamond. *Phys. Rev. B*, 80:075419, Aug 2009.

[8] José MP Nascimento and José MB Dias. Vertex component analysis: A fast algorithm to unmix hyperspectral data. *IEEE transactions on Geoscience and Remote Sensing*, 43(4):898–910, 2005.

[9] Michael E Winter. N-findr: An algorithm for fast autonomous spectral end-member determination in hyperspectral data. In *Imaging spectrometry V*, volume 3753, pages 266–275. SPIE, 1999.

[10] Hervé Abdi and Lynne J Williams. Principal component analysis. *Wiley interdisciplinary reviews: computational statistics*, 2(4):433–459, 2010.
